# Small extracellular vesicles deliver osteolytic effectors and mediate cancer‐induced osteolysis in bone metastatic niche

**DOI:** 10.1002/jev2.12068

**Published:** 2021-02-18

**Authors:** Qinyu Ma, Mengmeng Liang, Yutong Wu, Ce Dou, Jianzhong Xu, Shiwu Dong, Fei Luo

**Affiliations:** ^1^ Department of Orthopedics Southwest Hospital Third Military Medical University Chongqing 400038 China; ^2^ Department of Biomedical Materials Science Third Military Medical University Chongqing 400038 China; ^3^ State Key Laboratory of Trauma Burns and Combined Injury Third Military Medical University Chongqing 400038 China

**Keywords:** bone metastatic niche, cancer‐induced osteolysis, extracellular vesicles, miRNAs

## Abstract

Extracellular vesicles (EVs) play critical roles in regulating bone metastatic microenvironment through mediating intercellular crosstalks. However, little is known about the contribution of EVs derived from cancer cells to the vicious cycle of bone metastasis. Here, we report a direct regulatory mode between tumour cells and osteoclasts in metastatic niche of prostate cancer via vesicular miRNAs transfer. Combined analysis of miRNAs profiles both in tumour‐derived small EVs (sEVs) and osteoclasts identified miR‐152‐3p as a potential osteolytic molecule. sEVs were enriched in miR‐152‐3p, which targets osteoclastogenic regulator MAFB. Blocking miR‐152‐3p in sEVs upregulated the expression of MAFB and impaired osteoclastogenesis in vitro. In vivo experiments of xenograft mouse model found that blocking of miR‐152‐3p in sEVs significantly slowed down the loss of trabecular architecture, while systemic inhibition of miR‐152‐3p using antagomir‐152‐3p reduced the osteolytic lesions of cortical bone while preserving basic trabecular architecture. Our findings suggest that miR‐152‐3p carried by prostate cancer‐derived sEVs deliver osteolytic signals from tumour cells to osteoclasts, facilitating osteolytic progression in bone metastasis.

## 1 INTRODUCTION

More than 90% of patients with metastatic prostate cancer will develop bone metastases and result in pathological bone destruction (Coleman, 1997). Molecular mechanism mediating the crosstalks between prostate cancer cells and bone cells leading to imbalanced bone remodelling has been an emphasis of recent research (Kim et al., 2020). Bone homeostasis is maintained and regulated by osteoblastic bone formation and osteoclastic bone resorption. In bone metastatic niche, tumour cells interplay with osteoclasts and osteoblasts leading to abnormal bone remodelling featured by ‘the vicious cycle’ (Croucher et al., 2016; Fornetti et al., 2018). Prostate cancer cells secrete soluble factors such as insulin‐like growth factor (IGF), platelet‐derived growth factor (PDGF), bone morphogenetic protein (BMP), Endothelin‐1 and PTHrP to promote osteoblast proliferation and differentiation, subsequently osteoblasts generate receptor activator of nuclear factor κB ligand (RANKL) and interleukin 6 (IL‐6) to activate osteoclastic bone resorption leading to the release of bone matrix‐embedded transforming growth factor beta (TGF‐β) and epidermal growth factor (EGF) to promote tumour growth (Fournier et al., 2015; Tang et al., 2009; Zhang, 2019).

The bone resorbing osteoclasts play essential roles in both physiological and pathological bone remodelling (Roodman, 2004; Schett & Gravallese, 2012; Teitelbaum, 2000). Two essential factors RANKL and macrophage‐colony stimulating factor (M‐CSF) are responsible for osteoclast differentiation (Boyle et al., 2003). In bone metastatic niche, osteoclastogenesis can be stimulated through diverse non‐canonical ways. Studies suggested that breast cancer cells secrete osteolytic factors such as interleukin (IL)‐1, IL‐6 and IL‐8, connective tissue growth factor (CTGF) and matrix metallopeptidase (MMP)‐1 leading to exacerbation of osteolysis (Kim et al., 2020; Sabokbar et al., 2016). Our group also showed that breast cancer‐derived IL‐11 promotes osteoclast differentiation in a RANKL‐independent way, which leads to bone matrix degradation and the occurrence of early osteolysis (Liang et al., 2019). Studies reported that prostate cancer cells can directly produce pro‐osteoclastogenic factors including RANKL and IL‐6 (Keller & Brown, 2004; Sottnik & Keller, 2013). Moreover, it has already been proved that osteoclast is an effective target in treating and preventing prostate cancer bone metastases (Smith, 2008). However, monoclonal antibody denosumab neutralizing RANKL treatment is not satisfactory in reducing tumour burden (Helo et al., 2012), suggesting that more molecular insights of interplays between prostate cancer cells and osteoclasts in metastatic niche need to be clarified.

Extracellular vesicles (EVs) comprise small EVs (sEVs, < 200 nm) and large EVs (lEVs, > 200 nm) with specific lipid bilayer membrane structure and contain amounts of non‐coding RNAs (Van Niel et al., 2018). Cargos of sEVs can be transferred from parental cell to recipient cell to serve as intercellular communication messengers (Mathieu et al., 2019). sEV‐mediated microRNAs (miRNAs) transfer is widely considered to be involved in the development of many cancers. MiRNAs are small non‐coding RNAs that can bind to the 3 'UTR of target mRNAs to suppress their transcription activity or facilitate mRNAs degradation (Ambros, 2004; Bartel, 2004). A growing number of studies have shown that multiple miRNAs regulate osteoclast differentiation (Tang et al., 2014). Our previous studies demonstrated that miR‐7b plays important roles in regulating osteoclast fusion in bone remodelling (Dou et al., 2018). In tumour microenvironment, cancer cells release large amounts of sEVs containing miRNAs and subsequently affect surrounding cells (Xu et al., 2018). Recently, cancer‐derived sEVs have been demonstrated to be involved in tumour‐associated angiogenesis, vascular permeability and recruitment of bone marrow progenitor cell which initiate pre‐metastasis niche formation (Guo et al., 2019; Peinado et al., 2011). A recent study reported that cancer‐derived miR‐940‐containing sEVs induce osteogenic differentiation and breed an osteoblastic phenotype in the bone metastatic niche (Croset et al., 2018). Another study showed that Cavin‐1 alters prostate cancer‐derived EV content and regulates EV‐mediated osteoclastogenesis and osteoblast proliferation (Inder et al., 2014). Morhayim et al. reported that the mineralization stage‐specific protein content of osteoblast‐secreted EVs communicate with prostate cancer stimulating tumour growth (Morhayim et al., 2015). However, it is unclear whether prostate cancer‐derived sEVs affect osteoclastic function and the development of osteolytic phenotype.

This study aimed to uncover the contribution of miRNAs cargo in sEVs derived from prostate cancer cells in regulating osteoclastogenesis and osteolytic progression. Using small RNA sequencing, we identified distinctive miRNA signatures in sEVs from osteolytic cell line PC3 (PC3/sEVs) and non‐osteolytic cell line C4 (C4/sEVs) and C4‐2 (C4‐2/sEVs). Further bioinformatics analysis and in vitro tests revealed that miR‐152‐3p expression is enriched in osteolytic PC3 cells and their cognate sEVs, and can be transferred to the recipient bone marrow macrophages (BMMs). We correlated the miR‐152‐3p expression with osteoclastogenenic activity and assessed its interactions with negative regulators involved in suppressing osteoclastogenesis. Finally, we tested the relative contribution of sEVs in osteolytic progression, and explored the potential of miR‐152‐3p as a therapeutic target to intervene the progression of prostate cancer‐induced osteolysis.

## 2 MATERIALS AND METHODS

### 2.1 Reagents

Primary cultured BMMs were used to mimic in vivo osteoclast differentiation. Mouse bone marrow cells were isolated from 11‐week‐old male C57BL/6 mouse hind limbs (femur and tibia) and incubated with M‐CSF (50 ng/ml) for 96 h to obtain BMMs. Human prostate cancer cell lines PC3, C4 and C4‐2, and mouse leukemic monocyte macrophage RAW264.7 cells were obtained from the American Type Culture Collection (Rockville, MD, USA). BMMs and PC3 were cultured with Dulbecco's Modified Eagle Medium (DMEM; Hyclone, Thermo Scientific, MA, USA) containing 10% foetal bovine serum (FBS; CELLCOOK, Guangzhou, China) and 1% penicillin‐streptomycin (Gibco, Thermo Scientific, MA, USA). C4 and C4‐2 were cultured with RPMI 1640 medium (1640, Gibco, Thermo Scientific, MA, USA) containing 10% FBS and 1% penicillin‐streptomycin. All cells were cultured in a 37℃ incubator containing 5% CO_2_‐enriched atmosphere. Recombinant mouse RANKL and recombinant mouse M‐CSF were purchased from R&D Systems (Minneapolis, MN, USA). TRAP stain kit was obtained from Sigma‐Aldrich (NY, USA). Annexin V and PI Apoptosis Kit (F6012) was purchased from US Everbright Inc. (Suzhou, China). Annexin V was purchased from BD Biosciences (New Jersey, Franklin Lake, USA), GW4869 was purchased from Med Chem Express (New Jersey, USA). Antibodies against Histone 3 (bs‐17422R), CD9 (bs‐2489R), CD81 (bs‐2489R), CD63 (bs‐23032R), NFATC1 (bs‐1417R), CTSK (bs‐1611R), c‐fos (bs‐10172R), MITF (bs‐1990R), TSG101 (bs‐1365R), LaminA/C (bs‐1839R), and β‐actin (bs‐0061R) were purchased from Bioss Antibodies (Beijing, China). Antibody against MAFB (#41019) was purchased from Cell Signaling Technology (Shanghai, China), antibody against Argonaute‐2 (ab32381) was obtained from Abcam (Cambridge, UK), while antibody against Alix (611621) was obtained from BD Biosciences.

### 2.2 Separation, characterization and quantification of EVs

We separated and characterized the structure and content of sEVs according to Minimal Information 2018 for Studies of Extracellular Vesicles (MISEV2018) guidelines proposed by the International Society for Extracellular Vesicles (ISEV) (Théry et al., 2018). Specifically, 1) we regarded the EV nomenclature in accordance with MISEV2018 guidelines; 2) we determined that the osteolytic function is specific to sEVs not a common function shared by both sEVs and lEVs; 3) we characterized and quantified the separated sEVs through transmission electron microscopy, western blot analysis of well‐established pan‐EV markers (CD81, CD63, Alix and TSG101), and nanoparticle tracking analysis; 4) we confirmed that miR‐152‐3p is a bona fide cargo of sEVs, it is not affected by RNA‐binding proteins such as Argonaute‐2; 5) to evaluate the contribution of specific components in EVs, we directly engineered EVs cargos through EV transfection, instead of altered the miRNAs expression in EV donor cells; 6) before we used GW4869 to decrease EVs production, we evaluated the cytotoxicity of GW4869 to cancer cells and its effect on osteoclastogenesis.

Prostate cancer‐derived sEVs were collected by differential centrifugations as previously described with some modifications (Mensà et al., 2020). Briefly, prostate cancer cells were cultured in EV‐free medium for 24 h before sEVs isolation. EV‐free medium is consisted of DMEM, 10% EV‐depleted FBS and 1% penicillin‐streptomycin. EV‐depleted FBS was either produced in‐house by ultracentrifugation of serum at 110,000 g for 16 h (SW 32 Ti rotor, k‐factor: 231.2, 30056 rpm) followed by sterile filtration through a 0.2 μm filter (Millipore) or purchased from Systembio (Cat.no EXO‐FBS‐50A‐1). The supernatant from 10^7^ donor cells was collected and centrifuged at 1000 g for 15 min and 3000 g for 15 min to remove cell debris and apoptotic bodies. Subsequently, the supernatant was centrifuged at 18,000 g for 30 min to further remove lEVs. sEVs were pelleted via ultracentrifugation at 110,000 g for 70 min. The pelleted sEVs were washed in 1 × phosphate‐buffered saline (PBS;Hyclone, Thermo Scientific, MA, USA) and centrifuged again. For sucrose cushion ultracentrifugation, after a single wash step described above, the pelleted sEVs were resuspended in control DMEM without PBS or penicillin‐streptomycin. sEV‐contained DMEM was loaded over 30% sucrose solution (prepared in D_2_O) slowly without mixing the two layers, and centrifuged at 110,000 g, 4°C for 2 h (SW 32 Ti rotor, k‐factor: 231.2, 30056 rpm). The EV‐depleted DMEM supernatant was collected and the sucrose layer was resuspended in 1 × PBS and ultracentrifuged at 110,000 g, 4°C for 70 min (Type 70 Ti rotor, k‐factor: 143.9, 38698 rpm) to pellet down the sEVs. Finally, the sEVs were resuspended in PBS and waited for further experiments. Ultracentrifugation operations were conducted using an Optima XE‐90 (Beckman Coulter) at 4°C. For transmission electron microscopy, the separated sEVs were diluted with PBS and then fixed in 2% paraformaldehyde. Then, the samples were applied to formvar copper grids and stained with uranyl acetate (Electron Microscopy Sciences, USA) for 10 min at room temperature. Samples were naturally dried and observed in a FEI Tecnai 110 kV microscope at an accelerating voltage of 80 kV, digital images were obtained. Nanoparticle tracking analysis measurements were performed with a NanoSight NS300 (NanoSight, UK). The EV samples were diluted to reach the optimal concentration for detection (20–40 particles/frame), three 60‐s videos were recorded of each sample, with the camera level and detection threshold both set at 10. Data were expressed as mean ± SD of the three replications. All measurements were performed at room temperature.

### 2.3 Small RNA‐seq analysis

Total RNA was isolated by using RNeasy mini kit (Qiagen, Germany). Paired‐end libraries were synthesized by using the TruSeq Small RNA Sample Preparation Kit (Illumina, USA) following TruSeq Small RNA Sample Preparation Guide. The library construction and sequencing were performed at Shanghai Sinomics Corporation. All raw data were uploaded to GEO database (accession number: GSE161419).

### 2.4 Induction of osteoclastogenesis

DMEM or 1640 consists of 10% FBS, and 1% penicillin‐streptomycin as complete medium for culturing BMMs. 50 ng/ml M‐CSF, 25 ng/ml RANKL were added to complete medium to prepare osteoclast induction medium. For culturing BMMs with sEVs, separated sEVs were added into induction medium (10^6^ sEVs per ml) and incubated with BMMs for 4 days, the medium was replaced every 2 days. Specifically, every 10^4^ BMMs were incubated with 5 × 10^5^ sEVs. As for PC3‐CM, PC3 were cultured in EV‐free medium for 24 h before the supernatant was collected, and the supernatant was added to the induction medium at a ratio of 1:4 to generate PC3‐CM. To deplete EVs in CM, supernatant was centrifuged at 18,000 g for 30 min to remove lEVs and 110,000 g for 70 min to remove sEVs. Supernatant lacking lEVs was added into the induction medium (1:4) to generate lEV‐free PC3‐CM, while supernatant depleted both lEVs and sEVs was added into induction medium (1:4) to generate EV‐free PC3‐CM. As for GW4869 pretreatment, neutral sphingomyelinases inhibitor GW4869 (Med Chem Express, HY‐19363, diluted to 10 μM) was added to the complete medium to incubate PC3 for 6 h followed by PBS washing for three times, then PC3 were cultured in EV‐free medium for 24 h before the supernatant was collected. BMMs (3 × 10^3^ cells per well) were seeded in 96‐well plates and cultured with complete medium for 24 h. Then the medium was replaced with induction medium containing sEVs or PC3‐CM to further induce osteoclastogenesis for 4 days. The culture medium was replaced every 2 days and cells were incubated at 37°C with 5% CO_2_. Using the same methods of cell culture after BMMs were seeded in 6‐well plates (1 × 10^5^ cells per well) and 24‐well plates (1 × 10^4^ cells per well). After induction with sEVs or CM, the proteins and RNAs of cells were collected for the next experiment.

### 2.5 Evaluation of osteoclastogenesis in vitro

In this study, we used TRAP staining and immunofluorescence staining of actin rings to evaluate osteoclastogenesis. For TRAP staining, cells were first washed three times with PBS after the culture medium was removed and fixed in 4% paraformaldehyde for 20 min, followed by stained with TRAP staining solution (0.1 mg/ml of naphthol phosphate disodium salt, 0.3 mg/ml of Fast Red Violet zinc chloride stain) according to the manufacturers’ instructions. After dyeing for 30 min, dye solution was washed off and the relative TRAP activity was analyzed by colorimetry. At the same time, the number of multinucleated osteoclasts (more than three nuclei) was analyzed and calculated by imageJ software. For immunofluorescence staining of actin rings, after cells were fixed by 4% paraformaldehyde for 20 min and permeabilized with 0.2% Triton X‐100 for 10 min. After blocking for 30 min, the antibody against vinculin (Sigma‐Aldrich, diluted to 1:500) was added to every well and incubated at 37°C for 1 h in the dark. Washed three times with PBS after incubation and added DAPI (1:1000) for 10 min to stain the nuclei in the dark. Finally, the cells were washed three times with PBS and observed using a confocal microscope (Zeiss, LSM‐880).

### 2.6 Transfection of EVs and cells using miR‐152‐3p mimics and inhibitors

We directly engineered miR‐152‐3p relative expression in sEVs using Exo‐Fect Exosome Transfection Kit (System Bio, USA) according to the manufacturer's instructions. Specifically, 120 μl sterile PBS containing 10^6^ sEVs, 10 μl Exo‐Fect solution, 20 μl miR‐152‐3p (20 pmol) mimics (MC12269, Thermo Fisher Scientific) or inhibitors (MH12269, Thermo Fisher Scientific) were mixed in a 1.5 ml eppendorf (EP) tube to form a 150 μl transfection system. Inverted the EP tube three times to mix the reaction system without vortexing and incubated the EP tube at 37°C for 10 min, then placed it on ice. Added 30 μl of ExoQuick‐TC to the system and inverted six times to mix well to stop the transfection reaction. After the EP tube was incubated on ice for 30 min, the supernatant was removed and the transfected sEVs were pelleted through centrifugation. The pelleted sEVs were resuspended in induction medium with a concentration of 10^6^ sEVs per ml. Specifically, every 10^4^ BMMs were incubated with 5 × 10^5^ sEVs for further experiments. Through the transfection of mimics (4464059, Thermo Fisher Scientific) or inhibitors (AM17010, Thermo Fisher Scientific) negative control miRNA, the control groups of transfected sEVs were established as described above.

To investigate the impact of miR‐152‐3p on osteoclastogenesis, BMMs were transfected with miR‐152‐3p mimics and inhibitors using Entranster‐R4000 (Engreen Biosystem, China). Briefly, 50 pmol miR‐152‐3p mimics or inhibitors were diluted and mixed into 25 μl DMEM, then 1 ul of EntransterTM‐R4000 was added to 24 μl DMEM at room temperature for 5 min. Afterwards, the above two diluents were mixed to generate a 50 μl reaction system for 15 min at room temperature. Every 50 μl of transfection solution was used to transfect a 24‐well plate BMMs (10^4^ cells per well).

### 2.7 Transfection of plasmid and lentivirus

For the *Mafb* rescue experiment, mouse *Mafb* expression plasmid lacking 3 'UTR was designed and constructed by GeneCopoeia (USA), and transfected into BMMs before sEVs incubation. Briefly, 1 μg plasmid, 0.75 μl Lipofectamine‐3000 (Invitrogen, USA) and P3000 regent (Invitrogen, USA) 2 μl were added into 50 μl complete medium. After mixed and incubated at room temperature for 15 min, mixture was added to 10^5^ BMMs for 24 h. Then, the transfected medium was replaced with complete medium contained 500 μg/ml selective antibiotic G418 (Gibco, Thermo Scientific, USA) for cell selection. The selection medium was changed every 2 days and maintained the G418 concentration until the stable transfected BMMs were selected.

For construction of luciferase‐expressing tumour cell line, 1 × 10^5^ tumour cells were seeded on 12‐well plates (per well). Multiplicity of infection (MOI)  = 20 was selected for transfection. Before infection, the medium was replaced with 500 μl complete medium. Then, 10 μl volume of virus stock (HBLV‐ZsGreen‐LUC‐PURO, 2 × 10^8^ TU/ml, Hanbio biotechnology, Shanghai, China) was added to each well. After 4 h of incubation, 500 ul of complete medium was added to continue the culture for 24 h. The virus‐containing culture medium was replaced with complete medium contained 2 μg/ml polybrene (Hanbio biotechnology, Shanghai, China) for cell selection. The selection medium was changed every 2 days and maintained the polybrene concentration until stable transfected tumour cells were selected.

### 2.8 Experimental animals

C57BL/6 mice and BALB/c nude mice were obtained from the Laboratory Animal Center of Third Military Medical University (Chongqing, CHINA). All experimental protocols were reviewed and approved by the Institutional Animal Care and Use Committee of Third Military Medical University. All mice were maintained under 12‐h light, 12‐h dark cycles and free to food and water, and the mice were euthanized according to the AVMA Guidelines for the Euthanasia of Animals.

For inoculation of prostate cancer cells, 6‐week‐old BALB/c nude mice were intratibially injected with 100 μl of PBS suspension containing 2 × 10^5^ tumour cells labelled with fluorescent dyes. PC3/sEVs pre‐transfected with miR‐152‐3p inhibitors (PC3/anti‐152‐3p‐sEVs) and inhibitors negative control (PC3/anti‐NC‐sEVs) were used for intravenously administration of mice every 2 days. At each administration, every 10^7^ sEVs (resuspended into 150 μl PBS) were intravenously injected into a mouse, while the sham group was intravenously injected with the same volume of PBS. The antagomir‐152‐3p and negative control (antagomir‐NC) were designed and obtained from GenePharma (Suzhou, China). For antagomir administration, antagomir‐152‐3p or antagomir‐NC were intravenously injected into mice (50 μg/g) every 2 days.

### 2.9 Bioluminescence imaging

200 mg/kg body weight of D‐luciferin was injected into mice by intraperitoneal injection 10 min before imaging. A total of 3% mixture of isoflurane (Central laboratory, Third Military Medical University, China) in oxygen was used for introductory anaesthesia and 1.5% mixture for maintenance anaesthesia. Xtreme (Bruker, Billerica, MA, USA) was used to detect the fluorescence emitted from the tumour, and the molecular imaging software (MI) version 7.2 was used to map and quantify the region of interest (ROI) of the tumour. Tumour sizes were recorded weekly and tumour volumes were calculated using formula length × width^2^ × 0.5.

### 2.10 Histological evaluation

Tibias were obtained from mice after euthanasia, and the bones were fixed in 10% neutral buffered formalin, then washed and decalcified in 10% EDTA solution for 2 weeks, and then embedded in paraffin. A microtome was used to slice the femur (10 μm) in the longitudinal direction for subsequent H&E staining and TRAP staining.

### 2.11 Statistical analysis

All data were representative of at least three experiments of similar results performed in triplicate unless otherwise indicated. The data were presented as means ± standard deviation. Comparisons between two groups were analyzed using independent unpaired two‐tailed Student's *t*‐tests, and comparisons between more than two groups were analyzed using one‐way ANOVA followed by Student‐Newman‐Keuls post hoc tests. The values were considered significant at *P* < 0.05. Statistically significant differences between the treatment and control groups are indicated as * (*P* < 0.05) or ** (*P* < 0.01). *N.S*. means no significant differences between two groups.

### 2.12 EV‐TRACK

We have submitted all relevant data of our experiments to the EV‐TRACK knowledgebase (EV‐TRACK ID: EV210003) 2017.

## 3 RESULTS

### 3.1 PC3‐derived sEVs promote osteoclastogenesis

**FIGURE 1:**
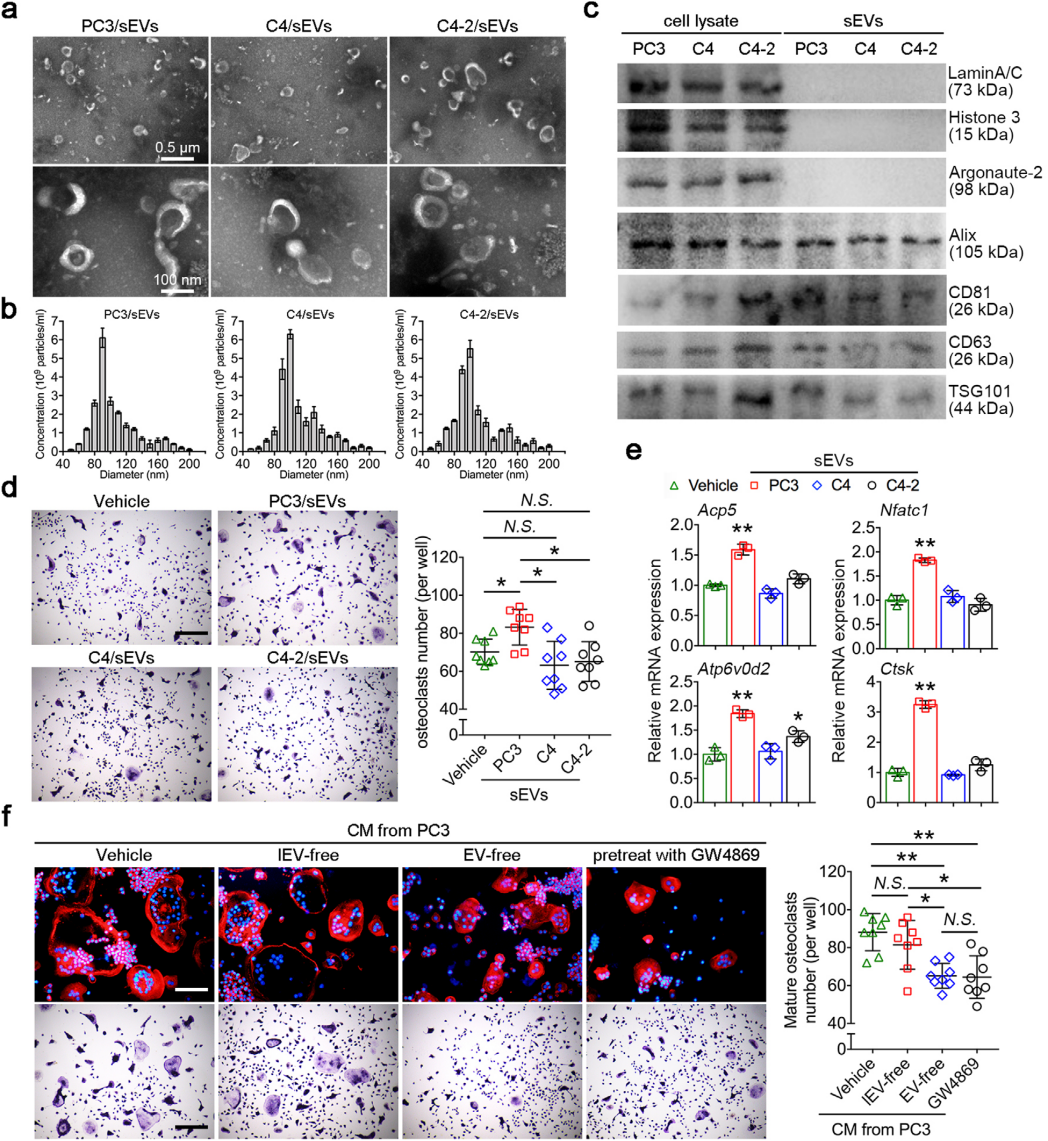
PC3‐derived sEVs promote osteoclastogenesis. (a) Transmission electron microscopy of sEVs derived three human prostate cancer cell lines: PC3/sEVs, C4/sEVs and C4‐2/sEVs. (b) Nanoparticle tracking analysis of PC3/sEVs, C4/sEVs and C4‐2/sEVs. (c) Western blot analysis showed the protein levels of LaminA/C, Histone 3, Argonaute‐2, Alix, CD81, CD63 and TSG101 in prostate cancer cells lysate and separated sEVs. (d) Representative TRAP staining images of BMMs cultured with PC3/sEVs, C4/sEVs and C4‐2/sEVs. Bar represents 400 μm. Quantification of multinucleated TRAP+ osteoclasts per well, *n* = 8. (e) Relative mRNA expression levels of *Acp5*, *Nfatc1*, *Atp6v0d2* and *Ctsk* in BMMs cultured with three sEVs, *n* = 3. (f) Representative actin ring and TRAP staining images of BMMs cultured with PC3‐CM depleted EVs using ultracentrifugation or GW4869. Bar represents 100 μm (top) and 400 μm (bottom). Quantification of multinucleated TRAP+ osteoclasts per well, *n* = 8. The data in the figures represent the averages ± SD. Statistically significant differences between the treatment and control groups are indicated as * (*P* < 0.05) or ** (*P* < 0.01). *N.S*. means no significant differences between two groups

To investigate the sEVs secreted from prostate cancer cells, we used three human prostate cancer cell lines: PC3, C4 and C4‐2. sEVs derived from these three prostate cancer cells were separated through a series of microfiltration and ultracentrifugation steps, followed by purification using sucrose cushion. Transmission electron microscopy analysis showed that sEVs derived from supernatant of PC3, C4, and C4‐2 were round shaped extracellular vesicles surrounded by lipid bilayer membranes (Figure 1 a). Nanoparticle tracking analysis showed that most vesicles in PC3/sEVs are ranged from 70 to 140 nm in diameter with a peak at 90 nm, whereas the majority of vesicles in C4/sEVs and C4‐2‐sEVs ranged from 70 to 150 nm in diameter with a peak at 100 nm (Figure 1 b). No significant differences were observed in particles/protein ratios between the three sEVs (Supplementary figure 1a). Western blot analysis of three sEVs revealed significant enrichment of classical EV markers including TSG101, CD81, Alix and CD63, as well as the absence of nuclear proteins such as LaminA/C, Histone 3 and Argonaute‐2 (Figure 1 c).

To explore the effect of prostate cancer‐secreted sEVs on osteoclast differentiation, BMMs were isolated from 11‐week‐old male C57BL/6 mouse hind limbs. BMMs were cultured with each kind of sEVs combined with 25 ng/ml RANKL stimulation for 96 h to generate osteoclasts, subsequently followed by TRAP staining (Figure 1 d). The results showed that PC3/sEVs have potential pro‐osteoclastic activity characterized by the highest osteoclast number with larger volume (Figure 1 d). PC3/sEVs treatment also upregulated the expression of nuclear factor of activated T‐cells cytoplasmic 1 (*Nfatc1*), cathepsin K (*Ctsk*), acid phosphatase 5 (*Acp5*) and *Atp6v0d2* in BMMs (Figure 1 e), any of which is a critical marker of osteoclastogenesis. We also confirmed that osteoclastogenesis substantially increased in the presence of PC3/sEVs in a dose‐dependent manner (Supplementary figure 1b, c). We further depleted EVs (lEVs and sEVs) in conditioned medium (CM) from PC3 (PC3‐CM), and used PC3‐CM to culture BMMs for 4 days of osteoclastogenesis. Depleting lEVs alone has no significant effect on osteoclastogenesis, while depletion of both lEVs and sEVs markedly reduced osteoclast number (Figure 1 f). GW4869 is a potent neutral sphingomyelinases inhibitor, which prevents the formation of intraluminal vesicles to further block sEVs production and release in numerous cell types. Treatment with GW4869 has no significant effect on both viability of PC3 and osteoclastogenesis, as confirmed by flowcytometry, CCK‐8 assay and TRAP staining (Supplementary figure 2a‐c). GW4869 pretreatment significantly reduced the secretion of sEVs from PC3, confirmed by NTA counting and western blot analysis of pan‐EV markers (Supplementary figure 2d, f). Consistently, the ratio of the number of secreted sEVs by per cell also reduced by 36.2% after GW4869 treatment as compared with control (Supplementary figure 2e). CM from PC3 pretreated with GW4869 also reduced osteoclast number compared with vehicle, approaching that seen by depleting lEVs and sEVs of PC3‐CM (Figure 1 f). Together, these data provide evidences that PC3/sEVs induced osteoclastogenesis, suggesting the potential involvement of sEVs in intercellular communication between tumour cells and osteoclasts in the bone‐tumour microenvironment.

### 3.2 miR‐152‐3p is a cargo of sEVs involved in osteoclastogenesis

**FIGURE 2:**
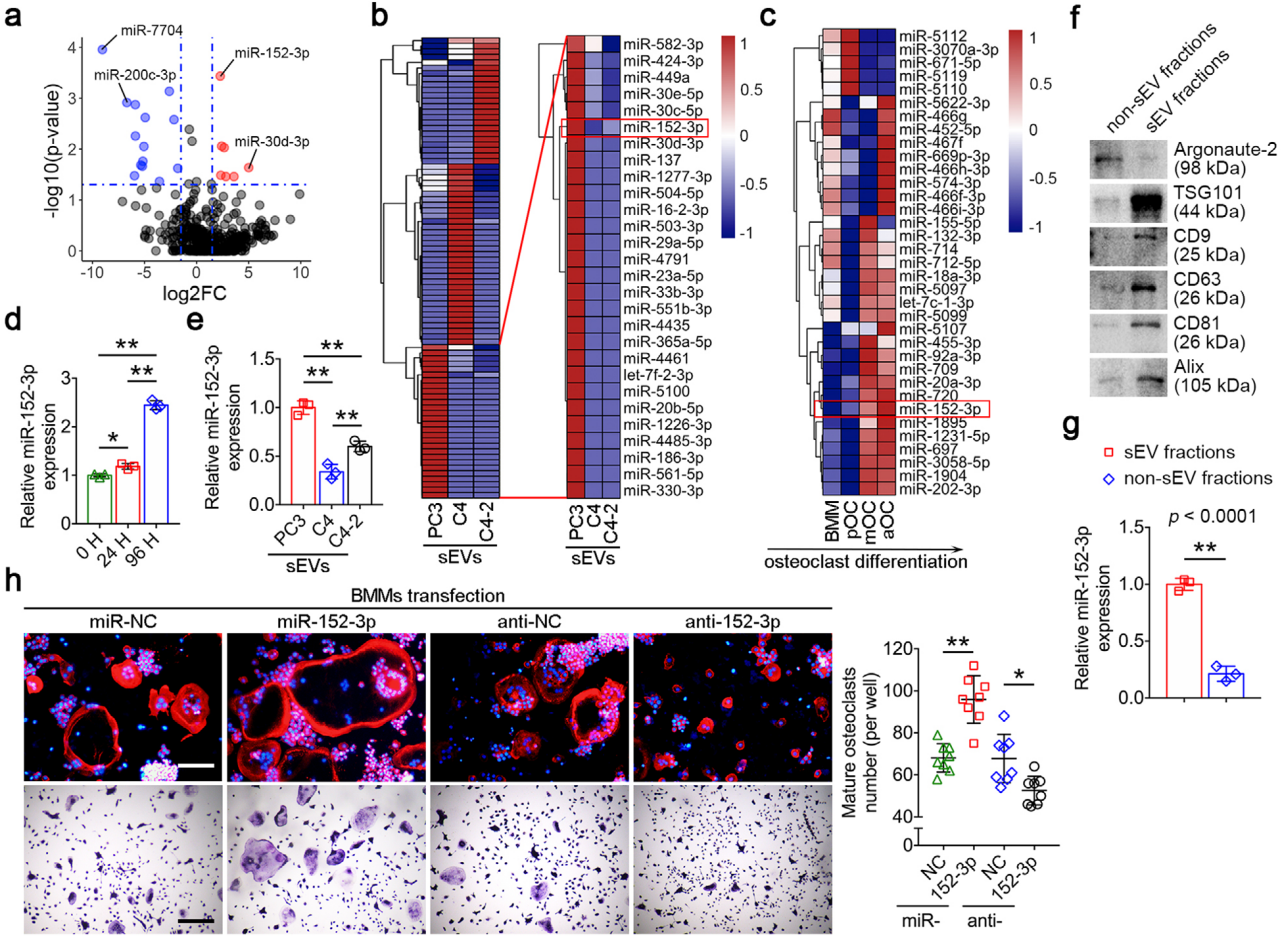
miR‐152‐3p is a cargo of sEVs involved in osteoclastogenesis. (a) Volcano plot showing differentially expressed miRNAs between two comparison groups (osteolytic PC3 *versus* non‐osteolytic C4 and C4‐2, log2FC > 1.5, ‐log10(*P*‐value) > 1.3). The average values of the miRNAs normalized expression of C4/sEVs and C4‐2/sEVs were used as a control for the miRNAs relative expression of PC3/sEVs. Red dots indicate upregulated miRNAs, while blue dots indicate downregulated miRNAs. (b) Clustering heatmaps showed the expression profile of miRNAs from three sEVs (left panel). The right panel showed the 28 miRNAs specifically enriched in PC3‐sEVs. Criteria: ‐log10(*P*‐value) > 1.3 and log2FC > 1. (c) A heatmap showed the miRNAs profile of osteoclast during osteoclastogenesis. (d) qRT‐PCR showed that the relative expression of miR‐152‐3p in BMMs constantly upregulated during osteoclastogenesis, *n* = 3. (e) Relative miR‐152‐3p expression in sEVs derived from three prostate cancer cell lines, *n* = 3. (f) Western blot analysis showed the protein levels of Argonaute‐2, TSG101, CD9, CD63, CD81 and Alix in EV fractions and non‐EV fractions after sucrose cushion ultracentrifugation. (g) Relative miR‐152‐3p expression in EV fractions and non‐EV fractions, *n* = 3. (h) Representative actin ring and TRAP staining images of BMMs transfected with miR‐152‐3p mimics, miR‐152‐3p mimics negative control, miR‐152‐3p inhibitors or miR‐152‐3p inhibitors control. Bar represents 100 μm. Quantification of multinucleated TRAP+ osteoclasts per well, *n* = 8. The data in the figures represent the averages ± SD. Statistically significant differences between the treatment and control groups are indicated as * (*P* < 0.05) or ** (*P* < 0.01)

Since miRNAs contained in EVs are demonstrated to play important roles in intercellular communications, we speculated that miRNAs contained in PC3/sEVs may play a role in affecting osteoclastogenesis. To explore further, we extracted total miRNAs of three sEVs and performed miRNA‐seq using Illumina NovaSeq 6000. Three prostate cancer‐derived sEVs were divided into two categories based on their different bone destruction phenotypes: 1) the osteolytic phenotype‐inducing cell lines PC3 and 2) the non‐osteolytic phenotype‐inducing cell lines (e.g., C4, C4‐2). A total of 772 miRNAs were detected, revealing 410 upregulated and 253 downregulated miRNAs with fold change > 2.0 across this group (Figure 2 a). A heatmap showed the top 84 miRNAs with most differential expression (Figure 2 b, left panel). As an initial step to identify miRNAs that may promote osteoclast differentiation, we focused on 28 novel miRNAs that significantly upregulated in sEVs from osteolytic PC3 cell lines (Figure 2 b, right panel). By analyzing our previously described gene expression data of BMM, preosteoclasts (pOC) and mature osteoclasts (mOC) during osteoclastogenesis (Dou et al., 2016), we identified a sustained upregulation of miR‐152‐3p during differentiation of BMM to osteoclasts, suggesting its potential involvement in regulating osteoclast differentiation (Figure 2 c). The expression of miR‐152‐3p during osteoclastogenesis, as well as in cancer cells and their cognate sEVs was further validated by qRT‐PCR (Figure 2 d, e, Supplementary Figure 3 a). After the purification of sucrose cushion, the sEV‐depleted sucrose solution after sucrose cushion purification was collected as non‐sEV fractions and compared with purified sEVs. Most pan‐EV markers were significantly enriched in sEV fractions compared with non‐sEV fractions, while Argonaute‐2 is enriched in non‐sEV fractions and is barely detected in sEV fractions (Figure 2 f). The relative miR‐152‐3p expression was significantly upregulated in sEV fractions compared with non‐sEV fractions (Figure 2 g). These results indicate that miR‐152‐3p is a bona fide cargo of sEVs.

To further explore the impact of miR‐152‐3p on osteoclast differentiation, mimics and inhibitors of miR‐152‐3p were used to transfect BMMs respectively, followed by TRAP staining and IF staining of actin ring to detect osteoclastogenesis. Transfection efficiency was confirmed by qRT‐PCR (Supplementary Figure 3b). After 4 days of osteoclast induction, TRAP staining revealed that miR‐152‐3p mimics significantly increased the number and size of osteoclasts, whereas transfection of miR‐152‐3p inhibitors resulted in a reduction in osteoclast number (Figure 2 h). We also confirmed the effects of miR‐152‐3p mimics and inhibitors on osteoclastogenesis at the mRNA level (Supplementary Figure 3c). Taken together, the combined analysis of multiple sequencing data screened out miR‐152‐3p may have an effect on osteoclast differentiation, and this biological effect was further confirmed by in vitro tests.

### 3.3 *Mafb* is a functional target of miR‐152‐3p

**FIGURE 3:**
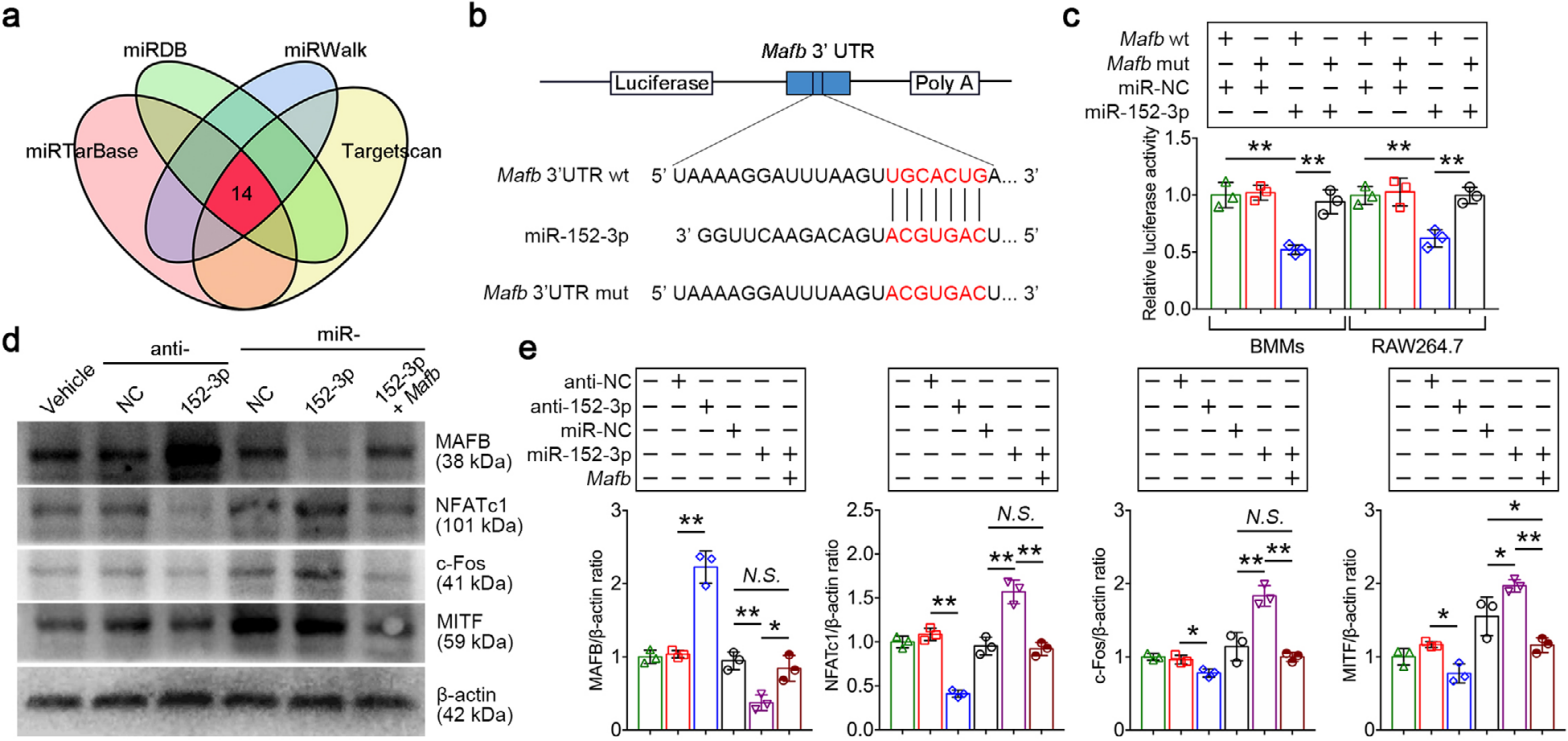
*Mafb* is a functional target of miR‐152‐3p. (a) VENN analysis showed that four target‐predicting algorithms were used to identify the potential downstream targets of miR‐152‐3p, *Mafb* was overlapped in all databases. (b) Construction of *Mafb* 3′ UTR wild type (wt) and mutant (mut) luciferase reporter vectors. (c) Relative luciferase activity of reporter containing the 3′ UTR of *Mafb* upon transfection with miR‐152‐3p mimics and mimics negative control in BMMs and RAW264.7 cells, *n* = 3. (d) Western blot analysis showed the protein levels of MAFB, NFATc1, c‐Fos, MITF and β‐actin in BMMs transfected miR‐152‐3p mimics, inhibitors or cotransfected with miR‐152‐3p mimics and *Mafb*. (e) Densitometric analysis of MAFB, NFATc1, c‐Fos, and MITF protein levels in indicated groups, *n* = 3. The data in the figures represent the averages ± SD. Statistically significant differences between the treatment and control groups are indicated as * (*P* < 0.05) or ** (*P* < 0.01). *N.S*. means no significant differences between two groups

To explore how miR‐152‐3p regulates osteoclastogenesis, we used four mRNA target‐predicting algorithms including miRTarBase, miRDB, miRWalk, and Targetscan to identify the potential downstream targets of miR‐152‐3p. Among all potential targets, *Mafb* was overlapped in all databases (Figure 3 a). It has been demonstrated that V‐maf musculoaponeurotic fibrosarcoma oncogene homolog B (MAFB, encoded by *Mafb*) interferes with the DNA binding ability of c‐Fos, MITF and NFATc1 thereby strongly inhibits osteoclastogenesis (Kim et al., 2007; Menéndez‐Gutiérrez et al., 2015). To establish whether *Mafb* is one of the targets of miR‐152‐3p, luciferase reporter plasmid containing the wild‐type 3′ UTRs of *Mafb* was generated and cotransfected with miR‐152‐3p mimics, while *Renilla* luciferase plasmid for normalization (Figure 3 b). Luciferase activities of *Mafb* were notably reduced upon the cotransfection of miR‐152‐3p mimics (Figure 3 c). On the protein level, western blot revealed that transfection of miR‐152‐3p inhibitors enhanced MAFB expression. Conversely, miR‐152‐3p mimics suppressed the expression levels of MAFB, whereas reintroduction of *Mafb* lacking the 3′ UTR regions restored the MAFB expression (Figure 3 d, e). We also detected the downstream molecules of MAFB including NFATc1, c‐Fos and MITF. Transfection of miR‐152‐3p mimics also significantly increased the expression of NFATc1, c‐Fos and MITF, while this effect was not reproducible by reintroduction of *Mafb* (Figure 3 d, e). Together, these results suggest that miR‐152‐3p plays a role in facilitating osteoclastogenesis through suppressing *Mafb* expressio

### 3.4 Prostate cancer‐derived miR‐152‐3p induces osteoclastogenesis through silencing *Mafb*

**FIGURE 4:**
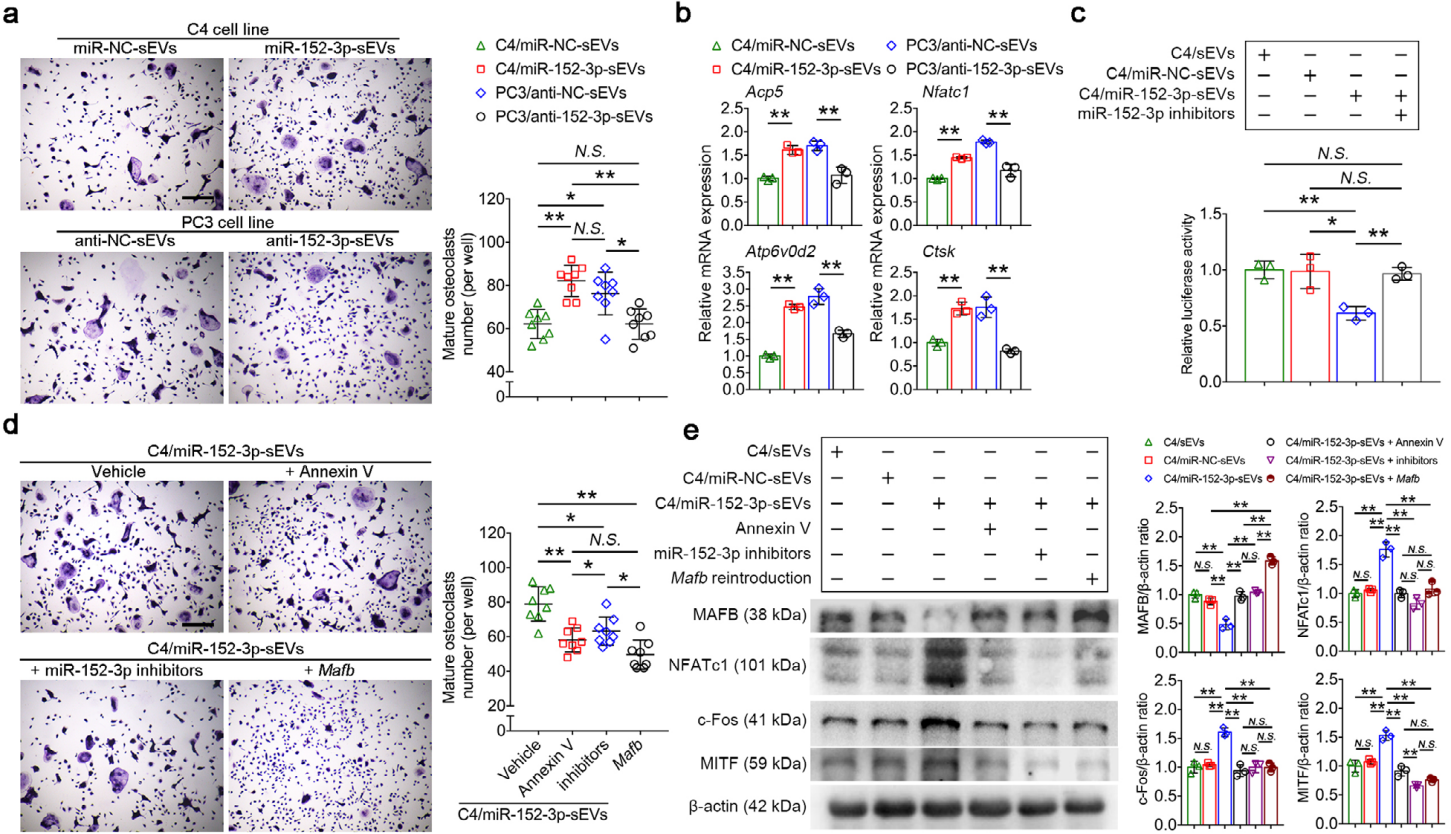
Prostate cancer‐derived miR‐152‐3p induces osteoclastogenesis through silencing *Mafb*. (a) Representative TRAP staining images of BMMs cultured with respective transfected sEVs. Bar represents 400 μm. Quantification of multinucleated TRAP+ osteoclasts per well, *n* = 8. (b) Relative mRNA expression levels of *Acp5*, *Nfatc1*, *Atp6v0d2* and *Ctsk* in BMMs cultured with respective transfected sEVs, *n* = 3. (c) Relative luciferase activity of the wild‐type *Mafb* 3′ UTR in BMMs was reduced by C4/miR‐152‐3p‐sEVs incubation, while transfection with miR‐152‐3p inhibitors abolished this effect, *n* = 3. (d) Representative images TRAP staining of BMMs cultured with C4/miR‐152‐3p‐sEVs. sEVs were pre‐incubated with Annexin V (2 μg/ml) or pretrasfected miR‐152‐3p inhibitors, recipient BMMs were restored *Mafb* expression through plasmid transfection. Bar represents 400 μm. Quantification of multinucleated TRAP+ osteoclasts per well, *n* = 8. (e) Western blot and densitometric analysis of MAFB, NFATc1, c‐Fos, MITF and β‐actin protein levels in BMMs in indicated groups, *n* = 3. The data in the figures represent the averages ± SD. Statistically significant differences between the treatment and control groups are indicated as * (*P* < 0.05) or ** (*P* < 0.01). *N.S*. means no significant differences between two groups

To further confirm the effect of miR‐152‐3p transferred by sEVs on osteoclastogenesis, sEVs from PC3 or C4 were transfected with miR‐152‐3p inhibitors or mimics, respectively. Transfection efficiency was confirmed by qRT‐PCR (Supplementary Figure 4 a). Transfection of miR‐152‐3p mimics and inhibitors did not affect the morphology and integrity of sEVs, as confirmed by transmission electron microscopy analysis (Supplementary Figure 4 b). Next, sEVs transfected with miR‐152‐3p inhibitors (PC3/anti‐152‐3p‐sEVs) and mimics (C4/miR‐152‐3p‐sEVs) were used to incubate BMMs for 72 h. qPCR analysis of sEV‐cultured BMMs revealed that miR‐152‐3p, but not primary miR‐152‐3p (pri‐miR‐152‐3p), was constantly upregulated in BMMs cultured with C4/miR‐152‐3p‐sEV, whereas miR‐152‐3p expression in PC3/anti‐152‐3p‐sEVs‐cultured BMMs showed no significant differences with time (Supplementary Figure 4 c, d). We further detected whether sEVs transfected with miR‐152‐3p mimics and inhibitors affected osteoclastogenesis, and the results showed that PC3/anti‐152‐3p‐sEVs significantly reduced osteoclastogenesis and downregulated the mRNA expression of *Nfatc1*, *Acp5*, *Ctsk* and *Atp6v0d2*. Conversely, C4/miR‐152‐3p‐sEVs increased the number of osteoclasts and upregulated the mRNA levels of osteoclast‐associated genes (Figure 4 a, b). These data suggested that prostate cancer‐derived sEVs promote osteoclastogenesis of BMMs via miR‐152‐3p transfer. We then examined the luciferase activities of the wild‐type *Mafb* 3′ UTR in BMMs cultured with C4/miR‐152‐3p‐sEVs. Results showed that culturing with C4/miR‐152‐3p‐sEVs significantly reduced the luciferase activities of *Mafb* compared with C4/miR‐NC‐sEVs or C4/sEVs, whereas application of miR‐152‐3p inhibitors abolished this effect (Figure 4 c). Annexin V is an inhibitor of sEVs internalization through blocking phosphatidylserine at the surface of sEVs (Li et al., 2013). TRAP staining revealed that treating sEVs with Annexin V (2 μg/ml) or transfecting miR‐152‐3p inhibitors in sEVs, or restoration of *Mafb* in recipient osteoclasts abolished the pro‐osteoclastic effect of C4/miR‐152‐3p‐sEVs, characterized by a significant reduction in osteoclast number (Figure  4 d). On the protein level, C4/miR‐152‐3p‐sEVs markedly downregulated the expression of MAFB and upregulated its downstream NFATc1, c‐Fos and MITF expression (Figure 4 e). Importantly, treatment with Annexin V or miR‐152‐3p inhibitors in C4/miR‐152‐3p‐sEVs abolished their suppression of MAFB, whereas restoration of *Mafb* in osteoclasts rescued MAFB expression and inhibited NFATc1, c‐Fos and MITF expression (Figure 4 e). These evidences suggest that miR‐152‐3p derived from sEVs is sufficient to promote osteoclastogenesis via targeting *Mafb*

### 3.5 PC3 increases tumour burden and induces the onset of osteolysis

**FIGURE 5:**
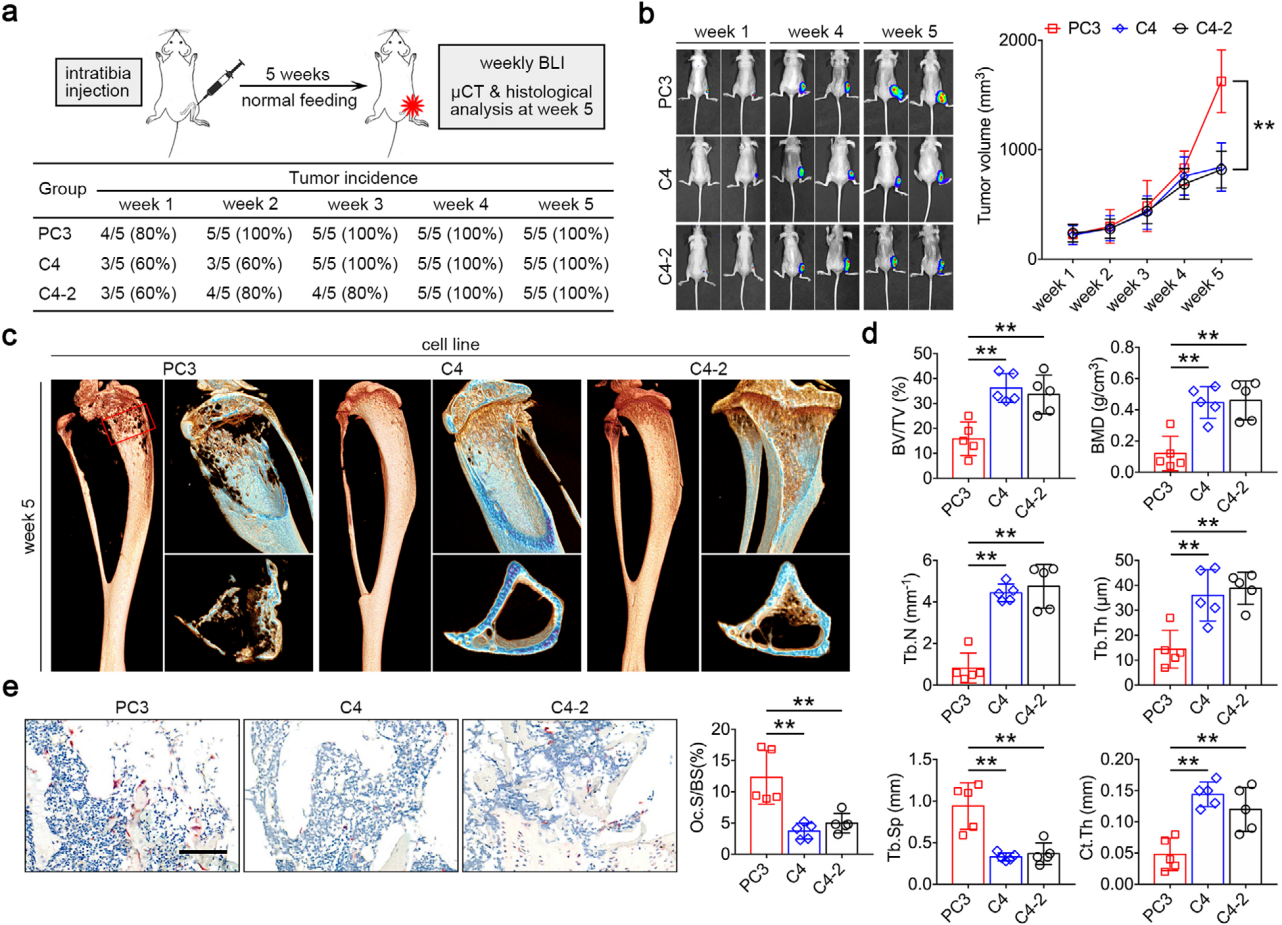
PC3 increases tumour burden and induces the onset of osteolysis. (a) Experimental design of xenograft mouse model through intratibia injection. (b) Representative BLI images and quantification of tumour volume size weekly, *n* = 5. (c) Representative micro‐CT images of tibias inoculated with three tumour cells at the week 5. (d) Quantification of trabecular bone volume fraction (BV/TV), bone mineral density (BMD), trabecular number (Tb.N), trabecular thickness (Tb.Th), trabecular separation (Tb.Sp) and cortical bone thickness (Ct.Th) of regions of interest in indicated groups, *n* = 5. (e) Representative histological TRAP images of tibia sections from mice inoculated with three tumour cells at the week 5, and quantification of osteoclast surface/bone surface. Bar represents 100 μm, *n* = 5. The data in the figures represent the averages ± SD. Statistically significant differences between the treatment and control groups are indicated as * (*P* < 0.05) or ** (*P* < 0.01)

To explore the role of miR‐152‐3p in intercellular crosstalks in tumour‐bone microenvironment, we established an orthotopic xenograft mouse model by injecting tumour cells into the intramedullary cavity of the tibia in BALB/c nude mice. This mouse model is useful to assess the interaction between implanted cancer cells and host cells in tumour‐bone microenvironment (Pang et al., 2020; Zhang et al., 2020). Bioluminescence imaging (BLI) was performed weekly using a firefly luciferase reporter stably expressed in the cell line to monitor the tumour progression in real time, while microcomputed tomography (micro‐CT) analysis was performed at the week 5 to evaluate the osteolytic destruction of tibias (Figure 5a). Among three human prostate cancer cell lines, no significant differences were observed in engraftment rate. However, PC3 inoculation induced more severe tumour burden in a relative late stage of cancer progression, while the growth of C4 and C4‐2 engraftment stopped increasing from the week 4 (Figure 5 b). Besides, PC3 also induced a typical osteolytic phenotype after a 5‐week inoculation characterized by reduced trabecular bone volume fraction (BV/TV) and bone mineral density (BMD), as confirmed by micro‐CT (Figure 5 c, d). To analyze the cortical and trabecular bone around the tibial plateau, we contoured an under 3 mm region beginning 0.8 mm proximal to the most proximal central epiphysis of the tibia. Trabecular separation (Tb.Sp) was significantly increased in mice inoculated PC3, whereas trabecular number (Tb.N), trabecular thickness (Tb.Th) and cortical bone thickness (Ct.Th) were decreased by PC3 injection in the week 5 (Figure 5c, d). These changes were rarely found in mice inoculated with C4 and C4‐2 cells. Histological TRAP staining of tibia sections revealed that the number of tumour‐induced osteoclasts significantly increased at bone‐tumour interface after PC3 inoculation, compared with the C4 or C4‐2 group (Figure 5 e). Generally, active osteolysis induced by cancer progression is accompanied by the release of massive TGF‐β from bone matrix (Crane & Cao, 2014; Juárez & Guise, 2011). Here we also performed ELISA to measure the TGF‐β expression in bone marrow and serum. Intriguingly, serum TGF‐β level of the PC3 group has no significant differences compared with the C4 or C4‐2 group from week 1 to 2, while it markedly enhanced at the week 3 and reached the highest level at the week 5. Whereas PC3 inoculation increased the TGF‐β level of bone marrow throughout the 5 weeks (Supplementary Figure 5). These results suggest that compared with non‐osteolytic C4 and C4‐2, PC3 induced typical osteolytic phenotype and more severe tumour burden.

### 3.6 Targeting miR‐152‐3p to rescue the osteolytic progression during tumour growth

**FIGURE 6:**
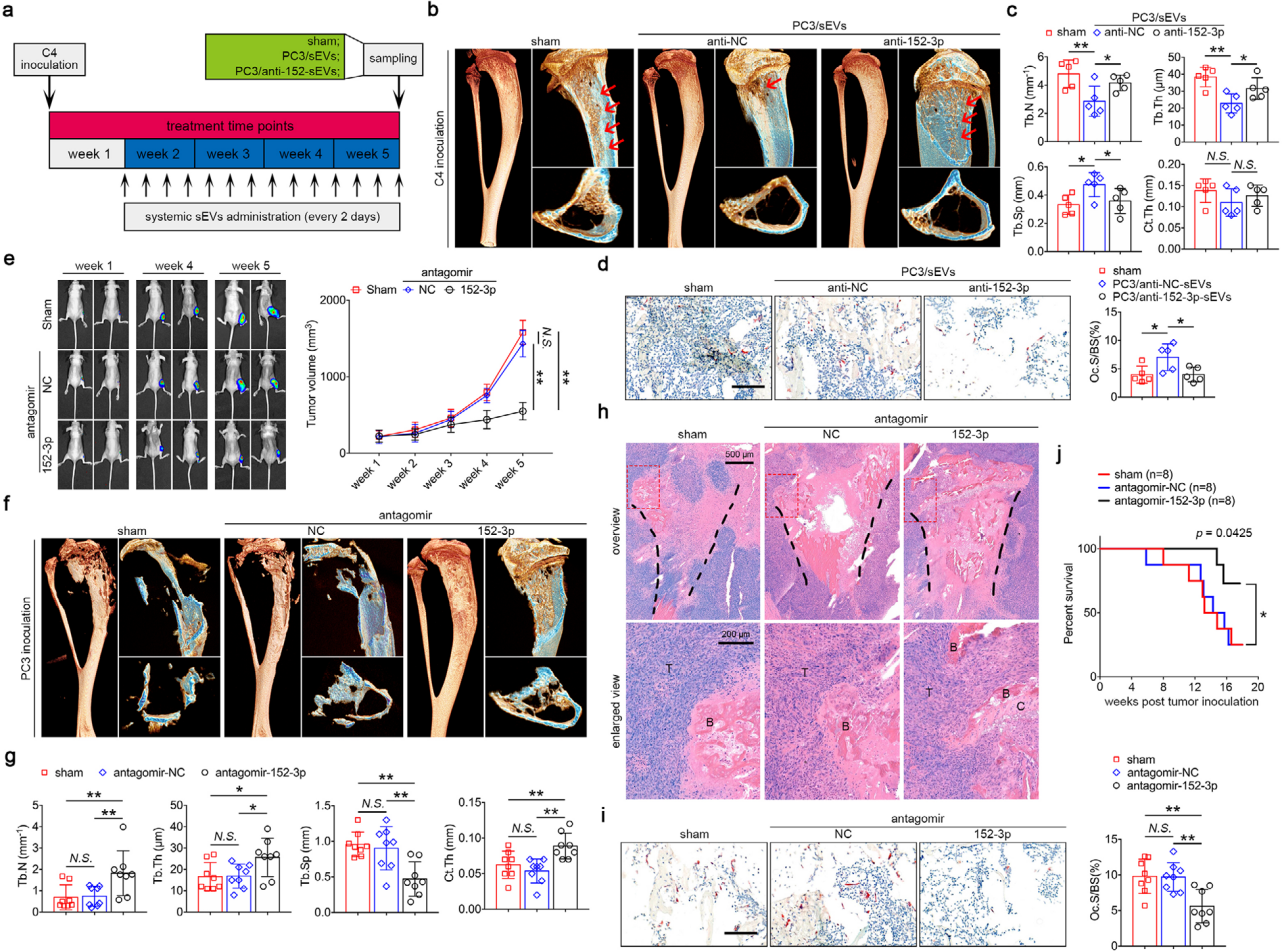
Targeting miR‐152‐3p to rescue osteolytic progression during tumour growth. (a) Xenograft mice inoculated with non‐osteolytic C4 were intravenously injected with PBS (150 μl), PC3/miR‐NC‐sEVs (1 × 10^7^, 150 μl) and PC3/anti‐miR‐152‐3p‐sEVs (1 × 10^7^, 150 μl) every 2 days from the week 1 till the week 5, *n* = 5. (b) Representative micro‐CT images of C4‐inoculated mice treated with PBS, PC3/anti‐NC‐sEVs and PC3/anti‐152‐3p‐sEVs. (c) Quantification of Tb.N, Tb.Th, Tb.Sp and Ct.Th in indicated groups, *n* = 5. (d) Representative histological TRAP images of tibia sections from mice treated with PBS, PC3/miR‐NC‐sEVs and PC3/anti‐miR‐152‐3p‐sEVs. Bar represents 100 μm. Quantification of osteoclast surface/bone surface in indicated groups, *n* = 5. (e) Representative BLI images and quantification of tumour volume size after antagomir‐152‐3p treatment, *n* = 8. (f) Representative micro‐CT images of PC3‐inoculated mice treated with PBS, antagomir negative control (50 μg/g) and antagomir‐152‐3p (50 μg/g) every 2 days until the week 5. (g) Quantitative analysis of Tb.N, Tb.Th, Tb.Sp and Ct.Th in indicated groups, *n* = 8. (h) Representative H&E staining of tibia sections from mice treated with three treatment groups. Bar represents 500 μm in overview and 200 μm in enlarged view. T, tumour; B, bone; C, cartilage. (i) Representative histological TRAP images of tibia sections from three treatment groups. Bar represents 100 μm. Quantification of osteoclast surface/bone surface in indicated groups, *n* = 8. (j) Kaplan‐Meier survival analysis of mice from three treatment group, *n* = 8. The data in the figures represent the averages ± SD. Statistically significant differences between the treatment and control groups are indicated as * (*P* < 0.05) or ** (*P* < 0.01). *N.S*. means no significant differences between two groups

To investigate whether sEVs affects osteolytic destruction in vivo, non‐osteolytic C4 cells were used to establish xenograft mouse model, followed by intravenously administration of PC3/sEVs every 2 days until the week 5 (Figure 6 a). Mice were examined by micro‐CT after 4 weeks of systemic sEVs administration, results revealed that treatment with PC3/sEVs did not change Ct.Th, but reduced Tb.N, Tb.Th and increased Tb.Sp compared with the sham group (Figure 6 b, c). However, transfection of miR‐152‐3p inhibitors in PC3/sEVs partly attenuated these effects characterized by improved trabecular architecture (Figure 6 b, c). The number of osteoclasts in bone‐tumour interface was significantly increased after PC3/sEVs administration, while this effect was not reproducible by transfection of miR‐152‐3p inhibitors (Figure 6 d). To test whether therapeutic inhibition of miR‐152‐3p can prevent tumour‐induced osteolysis, we used antagomir‐152‐3p for pulsed systemic administration in mice after 1‐week PC3 inoculation (Supplementary Figure 6a). As shown in Figure 6 e, treatment of antagomir‐152‐3p markedly reduces tumour burden from the week 4 (*P* = 0.0006), and this effect becomes more significant at the week 5 (*P* < 0.0001). Trabecular architecture was improved upon antagomir‐152‐3p administration, while mice treated with antagomir‐152‐3p had roughly two times more Tb.N than those in the antagomir‐NC group or the sham group (Figure 6 f, g). Histological analysis revealed that antagomir‐152‐3p treatment maintained the structural integrity of cortical bone, subchondral bone, growth plate and tibial epiphysis. In contrast, severe resorption of cortical bone and tumour invasion were observed in the sham and antagomir‐NC groups, with massive tumour cells occupying the marrow cavity (Figure 6 h). Abundant osteoclasts were observed around the surface of bone fragments in the sham and antagomir‐NC groups, whereas the number of tumour‐induced osteoclasts decreased significantly after antagomir‐152‐3p administration (Figure 6 i). The survival percentage was also increased in mice treated with antagomir‐152‐3p, compared with the sham or antagomir‐NC group (Figure 6 j). Since antagomir‐152‐3p has no significantly inhibitory effect on the apoptosis and proliferation of PC3 cells (Supplementary Figure 6b, c), this potent suppression of antagomir‐152‐3p in PC3‐induced osteolysis may be largely attributed to the intervention of osteoclastogenesis to block the vicious cycle in tumour‐bone microenvironment.

## 4 DISCUSSION

Tumour‐derived EVs are key factors regulating tumour microenvironment before and after bone metastasis. Tumour cells transfer EVs to surrounding cells and consequently alter their gene expression patterns to establish a favourable microenvironment for tumour cells survival and growth (Tkach & Théry, 2016). Vesicular communications between tumour cells and endothelial cells, tumour cells and mesenchymal cells mediate the formation of pre‐metastatic niche (Hoshino et al., 2015; Zeng et al., 2018). Similarly, growing evidences supported that EVs are involved in the crosstalks between tumour cells and adjacent bone cells in bone metastatic niche (Peinado et al., 2012; Tamura et al., 2020). In this study, we set out to investigate the contribution of prostate cancer‐derived sEVs in transferring osteolytic signals to osteoclasts. We used several cell lines of human prostate cancer for in vitro and in vivo investigation: PC3 was used as an osteolytic cell line, C4 and C4‐2 were non‐osteolytic cell lines used as controls. It is worth noting that the osteolytic properties of PC3 have been uncovered in recent years. PC3 induces osteoclastogenesis in various ways including CM, soluble factors and EVs in vitro (Fritz et al., 2007; Inder et al., 2014; Mizutani et al., 2009; Rafiei & Komarova, 2013). Xenograft mice inoculated with PC3 develop a typical osteolytic phenotype characterized by increased osteoclast‐mediated bone resorption and decreased bone formation (Alsulaiman et al., 2016; Fradet et al., 2013; Secondini et al., 2011). By contrast, C4 and C4‐2 have been shown to induce an osteoblastic destruction instead of osteolysis (Corey et al., 2005; Thalmann et al., 1994; Wu et al., 1998). Consistent with previous studies, we showed that both CM and EVs of PC3 have the pro‐osteoclastic properties in vitro and in vivo. One step further, we found that depleting lEVs in PC3‐CM did not affect its pro‐osteoclastic activity, whereas depletion of lEVs and sEVs reduced osteoclastogenesis, suggesting that the osteolytic properties of PC3 at the vesicular level are largely attributed to sEVs. These intriguing findings on functional differences also support the hypothesis that different EVs (sEVs and lEVs) shuttle different cargos (Mensà et al., 2020; Murillo et al., 2019).

Bone metastasis is a complex cascading process. Previous views suggested that the vicious cycle of prostate cancer‐induced osteolysis is mainly attributed to the fact that tumour cells induce the differentiation and RANKL release of osteoblasts, which lead to the excessive activation of osteoclasts (Wu et al., 2017; Zhang, 2019). Studies also showed that prostate cancer cells secrete cytokines that stimulate osteoclast differentiation (Keller & Brown, 2004; Sottnik & Keller, 2013). Intriguingly, we found that PC3/sEVs have the biological potential to directly promote osteoclastogenesis in vitro, while injecting osteolytic PC3/sEVs into xenograft mice inoculated with non‐osteolytic C4 resulted in the destruction of trabecular architecture. In this regard, our data supported the hypothesis that prostate cancer directly affects osteoclastogenesis and osteolytic progression in an EV‐mediated manner, and this interactive mode may mediate the acceleration of vicious cycle in bone metastasis (Maurizi & Rucci, 2018).

Although bone metastasis of prostate cancer is still lack of treatments, the development of drug therapy has allowed to partially reduce cancer progression and mortality rate (Sartor & De Bono, 2018). As an important contributor in the vicious cycle, osteoclast has received much attention regarding the treatment of prostate cancer bone metastasis. Inhibition of bone resorption, with either bisphosphonates or recombinant osteoprotegrin, alleviated osteolytic destruction, tumour burden and cancer‐induced bone pain, indicating the critical role of activated osteoclasts in tumour progression (Helo et al., 2012; Saad, 2004; Wu et al., 2019). In recent years, increasing studies were devoted to finding important contributors in cancer progression including specific cells types and molecules, thereby raising the possibility of searching new therapeutic targets (Cook et al., 2014; Lipton, 2006). In this study, miR‐152‐3p was identified as an osteolytic molecule contained in prostate cancer‐derived sEVs. Blocking miR‐152‐3p in PC3/sEVs abolished the effect of PC3/sEVs on trabecular architecture destruction, while antagomir‐152‐3p treatment preserved basic trabecular architecture and further reduced the osteoclast‐mediated cortical bone destruction. This direct regulation mode of prostate cancer cells on osteoclastogenesis provides a basis for exploring new therapeutic targets and methods in bone metastasis treatment.

miR‐152‐3p has been reported to be markedly upregulated in plasma from patients with prostate cancer, and is considered to be a potential biomarker for prostate cancer diagnosis (Moya et al., 2019). Besides, the expression of miR‐152‐3p is also thought to be correlated with cancer progression and prognosis (Chen et al., 2016; Moya et al., 2019). In bone, miR‐152‐3p is consistently upregulated in patients with postmenopausal osteoporosis and osteoporotic vertebral fractures (Kocijan et al., 2016; Zarecki et al., 2020). These evidences suggest that the expression level of miR‐152‐3p is closely associated with cancer progression and excessive osteoclast activation. Our findings further connected these clues by showing that prostate cancer‐derived sEVs were enriched in miR‐152‐3p, of which was capable of targeting *Mafb*, a negative regulator controlling osteoclastogenesis. Actually, the binding relationship between miR‐152‐3p and *Mafb* has been described in a recent study, the researchers showed that modulation of miR‐152‐3p/*Mafb* axis in microglia is involved in the development of neuropathic pain (Tozaki‐Saitoh et al., 2019). However, since the role of MAFB in microglia‐mediated pain and osteoclast‐mediated bone resorption is totally different (Kim et al., 2007; Menéndez‐Gutiérrez et al., 2015). Our data indicated the extensive stability of miR‐152‐3p/*Mafb* signaling in different cell types, paving the way for further research on this regulatory axis. Our in vivo studies showed that antagomir‐152‐3p treatment is effective in slowing the progression of osteolysis after bone metastasis. However, for clinical use, the miRNA market that is still in his infancy. The use of miRNA inhibitors or antagomir products is less advanced due to pharmacokinetic effectiveness and safety issues (Bonneau et al., 2019).

In conclusion, our findings lay the foundation for further research on cell‐cell interactions in bone metastatic niche, especially revealing how EVs transfer osteolytic signals from tumour cells to osteoclasts. At the same time, drugs targeting pro‐osteoclastic regulators and intervention of miRNAs expression also have considerable potentials to interfere with the progression of osteolytic lesions after bone metastasis.

## CONFLICTS OF INTEREST

The authors report no conflict of interest.

## AUTHOR CONTRIBUTIONS

Qinyu Ma designed study, performed experiments, analyzed data, wrote the initial draft of manuscript and revised manuscript. Mengmeng Liang prepared the EV samples for small RNA‐seq. Mengmeng Liang and Yutong Wu performed experiments and contributed to the revision of manuscript. Ce Dou contributed to the correction of manuscript. Jianzhong Xu provided financial support. Shiwu Dong supervised the progress of study, provided experimental platform and financial support. Fei Luo supervised the progress of study, provided financial support and critical review of study.

## Supporting information



Supplementary information
