# Supplementary material for: Small extracellular vesicles deliver osteolytic effectors and mediate cancer‐induced osteolysis in bone metastatic niche
Source: J Extracell Vesicles. 2021 Feb 18;10(4):e12068. doi: 10.1002/jev2.12068 (PMC7892803; doi:10.1002/jev2.12068)
Supplement: Supplementary file 1 — Supplementary information [file JEV2-10-e12068-s001.docx]

**Small extracellular vesicles deliver osteolytic effectors and mediate cancer-induced osteolysis in** **bone metastatic niche**

Qinyu Ma ^a*^, Mengmeng Liang ^b^, Yutong Wu ^a^, Ce Dou ^a^, Jianzhong Xu ^a^, Shiwu Dong ^b,c*^ and Fei Luo ^a*^

^a^ Department of Orthopedics, Southwest Hospital, Third Military Medical University, Chongqing 400038, China.

^b^ Department of Biomedical Materials Science, Third Military Medical University, Chongqing 400038, China.

^c^ State Key Laboratory of Trauma, Burns and Combined Injury, Third Military Medical University, Chongqing 400038, China.

**Corresponding author:**

Qinyu Ma, Department of Orthopedics, Southwest Hospital, Third Military Medical University, Gaotanyan Street No.30, Chongqing 400038, China Email: mqy32473069@gmail.com

Shiwu Dong, Department of Biomedical Materials Science, Third Military Medical University, Chongqing 400038, China. Email: dongshiwu@tmmu.edu.cn

Fei Luo, Department of Orthopedics, Southwest Hospital, Third Military Medical University, Gaotanyan Street No.30, Chongqing 400038, China. Email: luofly1009@hotmail.com

**Keywords:** extracellular vesicles; bone metastatic niche; cancer-induced osteolysis; miRNAs

**Supplementary Materials and Methods**

*Western blot analysis*

Protein samples extracted from cells or sEVs were lysed in radio immune precipitation assay buffer (Beyotime Biotechnology, Nantong, Jiangsu, China) containing a protease inhibitor cocktail and incubated on ice for 30 min. After centrifugation at 13000 g, the supernatant was carefully removed and transferred into a new microfuge tube. For ultrafiltration, the solution was concentrated to 500 μl in Amicon® Ultra-15 3KD devices (Cat.no UFC900324) using an Allegra® X-15R centrifuge at 4,000 g at 4°C for 30 min. The concentration of proteins was quantified using the Enhanced BCA [Protein Assay](https://www.sciencedirect.com/topics/biochemistry-genetics-and-molecular-biology/protein-assay) Kit (Beyotime Biotechnology, China). Each sample (50 μg) was diluted in loading buffer and subjected to a standard SDS-PAGE followed by transferred onto PVDF membranes (ImmobilonTM-PSQ Membranes, Sigma-Aldrich, Saint Louis, MO, USA). After blocking in 5% skim milk, proteins were detected using the following antibodies: Histone 3 (bs-17422R) at a 1:1000 dilution, LaminA/C (bs-1839R) at a 1:1000 dilution, CD81 (bs-2489R) at a 1:1000 dilution, CD9 (bs-2489R) at a 1:1000 dilution, CD63 (bs-23032R) at a 1:1000 dilution, TSG101 (bs-1365R) at a 1:1000 dilution, Argonaute-2 (ab32381) at a 1:1000 dilution, Alix (611621) at a 1:1000 dilution, NFATC1 (bs-1417R) at a 1:1000 dilution, c-fos (bs-10172R) at a 1:1000 dilution, MITF (bs-1990R) at a 1:1000 dilution, MAFB (#41019) at a 1:1000 dilution, and β-actin (bs-0061R) at a 1:2000 dilution. Corresponding secondary antibodies against primary antibodies were used by 1.5 hours of incubation at room temperature (1:2000). Blots against β-actin served as loading control. The protein level is colored by enhanced chemiluminescence (ECL, Bioground, China), and detected and imaged by ChemiDoc MP Imaging System (Bio-Rad, USA).

*Real-time PCR analysis*

RNA was extracted from cells or sEVs using Trizol reagent (Life Technologies, NY, USA). Total RNA was reverse transcribed into cDNA using a cDNA reverse transcription kit (ReverTra Ace qPCR RT Master Mix, TOYOBO, Japan) according to the manufacturer's instructions. SYBR Green Realtime PCR Master Mix (TOYOBO, Japan) was used for the real-time polymerase chain reactions and PCR products were detected on a CFX96 Real-Time PCR System instrument (Bio-Rad). Reaction conditions were as follows: step 1: 95 °C for 1 min, step 2: 40 cycles of 95 °C for 15 sec, 60 °C for 15 sec, 72 °C for 45 sec, step 3: melting curve analysis. U6 and GAPDH were used as internal controls for miRNAs and mRNAs, respectively. Specific primers are listed in Supplementary Table 1. The 2^−ΔΔCT^ method was used to calculate relative expression of miR-152-3p. The relative amount of miR-152-3p was calculated as ΔCt=Ct (miR-152-3p) −Ct (RNU-6). ΔCT value was then normalized to control samples (ΔΔCT), and the 2^−ΔΔCT^ value was obtained.

*Flowcytometry*

We conducted flow cytometric analysis using a BD Accuri C6 flow cytometer, counting 10000 events. After marking with AnnexinV-FITC and PI, the remaining binding buffer was removed and cells were pelleted by centrifugation at 300×g for 10 min, and resuspended in 500 μl PBS for flowcytometry analysis.

*μCT analysis*

After the xenograft mice were euthanized, the tumor legs were collected and put into a 5 ml centrifuge tube filled with 10% formalin solution. A Bruker MicroCT Skyscan 1272 system (Kontich, Belgium) was used to capture the images of the whole mouse tibia with an isotropic voxel size of 10.0 μm. Scanning was done using a 60 kV X-ray tube with an X-ray intensity of 166 μA at an exposure time of 1700 ms. For trabecular bone analysis of the tibial plateaus, an under 3 mm region beginning 0.8 mm proximal to the most proximal central epiphysis of the tibias was contoured. For cortical bone analysis of tibias (2D analysis), a 0.5 mm region beginning 4 mm proximal to the most proximal central epiphysis of the tibias. 3D reconstruction of the CT images was performed based on distance transformation of the grey scale original images (CTVox, Ver. 3.0.0). 3D and 2D analysis were performed using software CT Analyser (Ver. 1.15.4.0, Kontich, Belgium). All images presented are representative of the respective groups.

*TGF-β1 ELISA*

Serum and bone marrow were isolated from xenograft mice after euthanasia to detect the quantitative level of TGF-β1. According to the manufacturer’s instructions, the samples are tested by Mouse TGF-beta 1 Quantikine ELISA Kit (R&D systems, China) in triplicate.

*CCK-8 assay*

3 × 10^3^ cells were seeded in 96-well plates and incubated with GW4869 (10 μM) or antagomir-152-3p (150 nM) for 1 d, 2 d, and 3 d. According to the manufacturer’s instructions, cell viability was evaluated using a CCK-8 assay kit (HyClone) at 1 d, 2 d, and 3 d. The absorbance of the wells was measured at 450 nm in a 96-well plate reader, and cell viability was evaluated.

**Supplementary Figures**


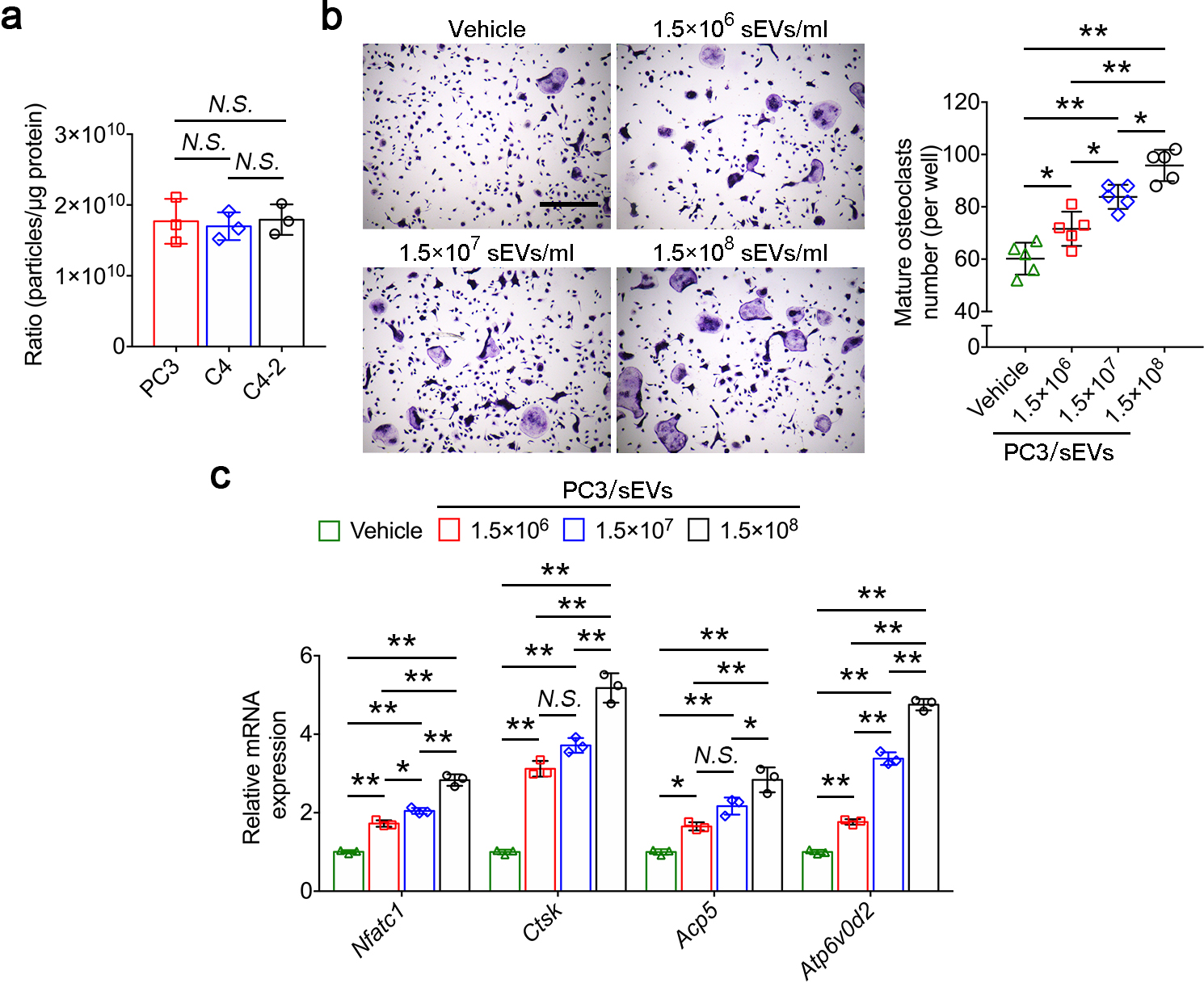


**Supplementary Figure S1. PC3-derived sEVs promote osteoclastogenesis**

(a) The ratio of particles/protein across three types of cancer cells were detected, n=3.

(b) Representative TRAP staining images of BMMs treated as indicated, n=5. Vehicle represents BMMs were cultured with induction medium without sEVs. Bar represents 500 μm.

(c) Relative mRNA expression levels of *Nfatc1*, *c-Fos*, *Atp6v0d2* and *Ctsk* in osteoclasts treated as indicated, n=3.

The data in the figures represent the averages ± SD. Statistically significant differences between the treatment and control groups are indicated as * (*p* < 0.05) or ** (*p* < 0.01). *N.S.* means no significant differences between two groups.


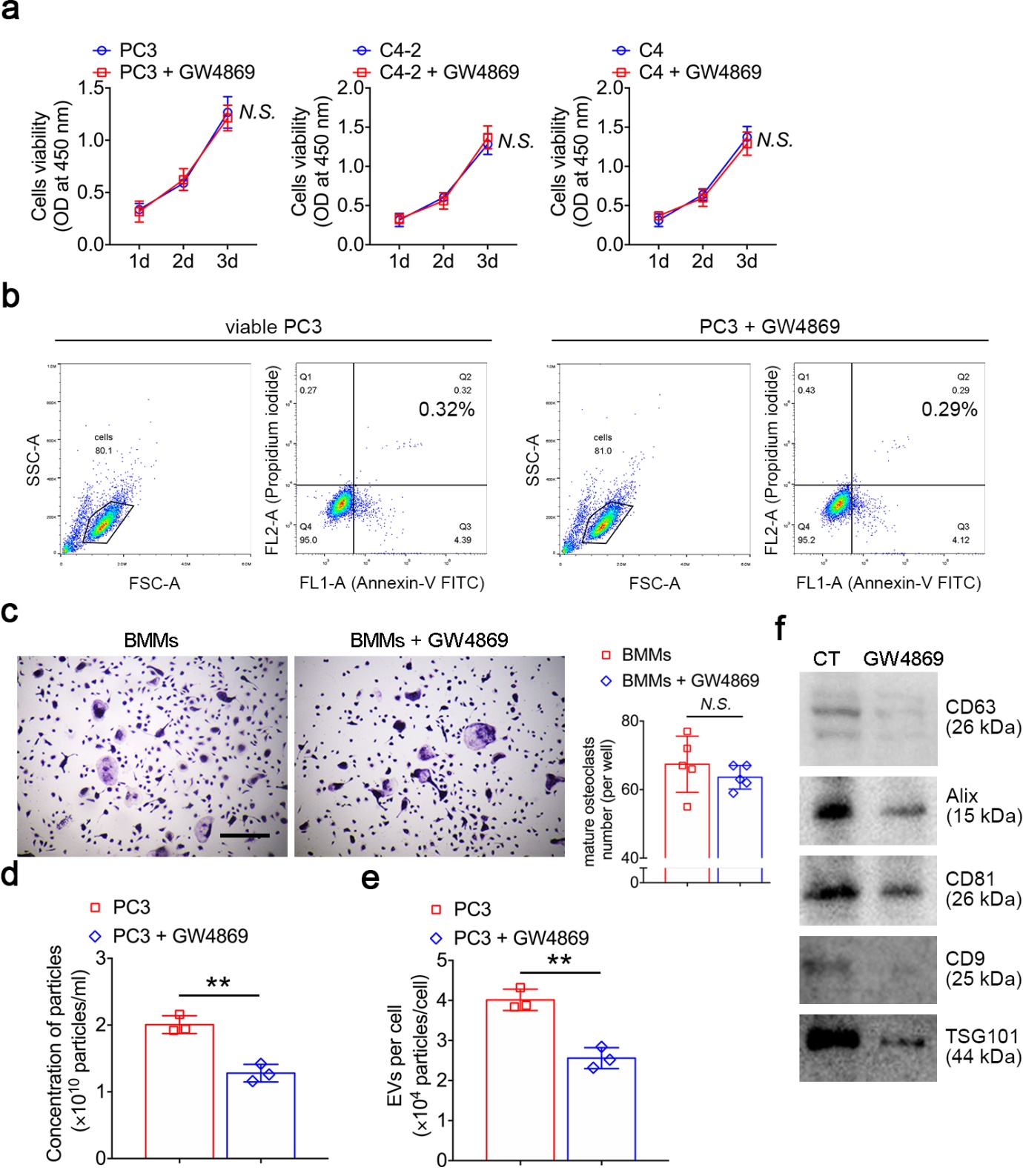


**Supplementary Figure S2. Cytotoxic evaluation of GW4869 on cancer cells and osteoclastogenesis**

(a) The viability of three types of cancer cells was assessed at 1, 2, and 3 days after treatment with GW4869.

(b) The cytotoxicity of GW4869 to PC3 was evaluated by flowcytometry.

(c) Representative TRAP staining images showed that GW4869 has no significant effects on osteoclastogenesis of BMMs. Bar represents 500 μm. Quantification of multinucleated TRAP+ osteoclasts per well, n=5.

(d) Concentration of particles derived from PC3 treated with GW4869, n=3.

(e) Ratio of the total number of sEVs per cell isolated from the supernatant of PC3 after treated with GW4869, n=3.

(f) Western blot analysis showed the protein levels of pan-EV markers in the supernatant of PC3 pretreated with GW4869, CT represents control group without GW4869 treatment.

The data in the figures represent the averages ± SD. Statistically significant differences between the treatment and control groups are indicated as * (*p* < 0.05) or ** (*p* < 0.01). *N.S.* means no significant differences between two groups.


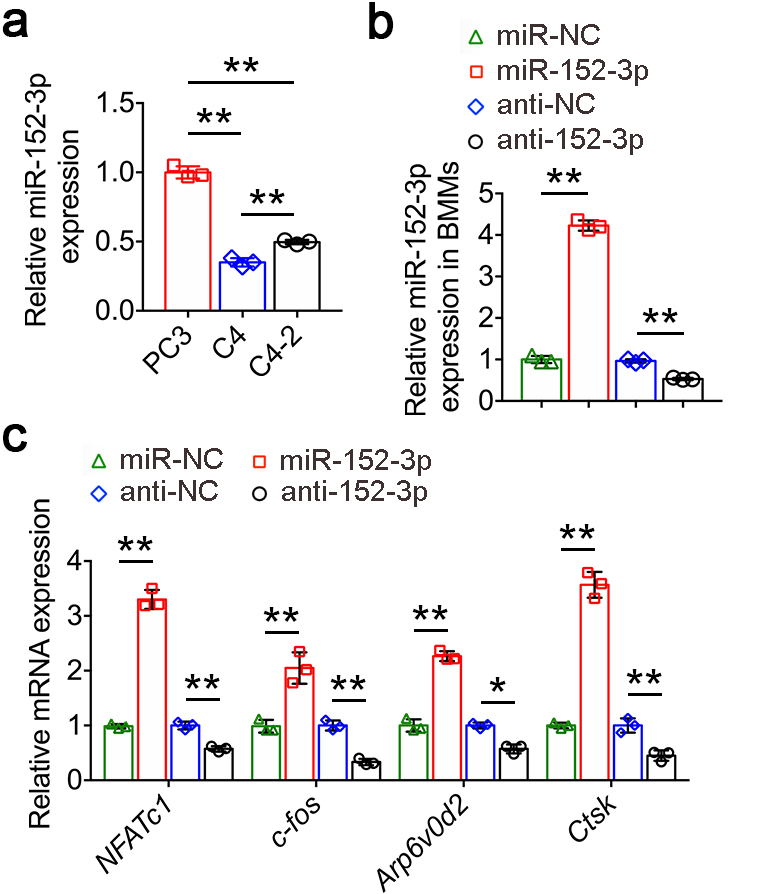


**Supplementary Figure S3. miR-152-3p is involved in osteoclastogenesis**

(a) Relative miR-152-3p expression was detected in three human prostate cancer cell lines, n=3.

(b) Relative miR-152-3p expression in BMMs transfected with miR-152-3p mimics and miR-152-3p inhibitors, respectively, n=3.

(c) Relative mRNA expression levels of *Nfatc1*, *c-Fos*, *Atp6v0d2* and *Ctsk* in osteoclasts transfected with miR-152-3p mimics and miR-152-3p inhibitors, respectively, n=3.

The data in the figures represent the averages ± SD. Statistically significant differences between the treatment and control groups are indicated as * (*p* < 0.05) or ** (*p* < 0.01). *N.S.* means no significant differences between two groups.


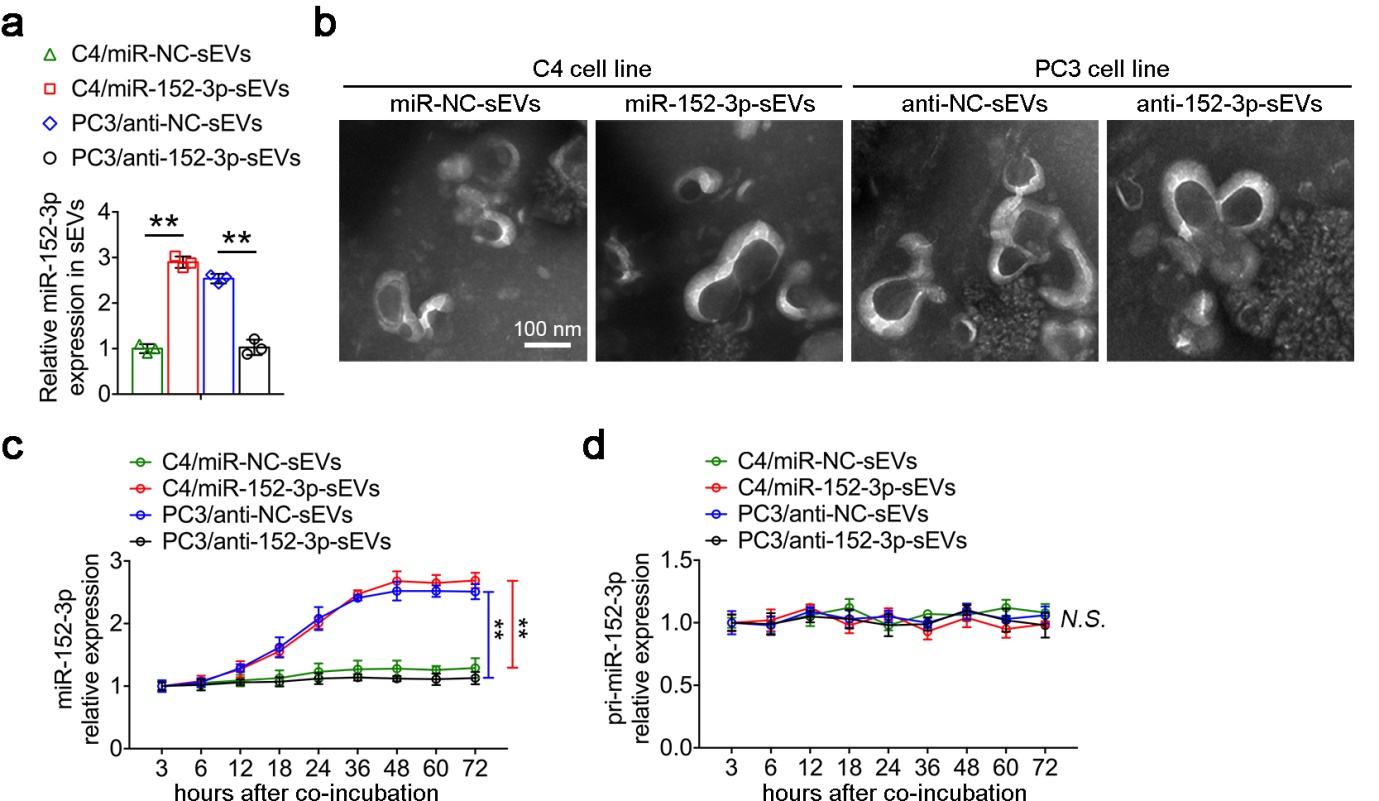


**Supplementary Figure S4. Prostate cancer-derived miR-152-3p induces osteoclastogenesis through silencing *Mafb***

(a) Relative miR-152-3p expression in sEVs transfected with miR-152-3p inhibitors or mimics, n=3.

(b) Transmission electron microscopy of sEVs transfected with miR-152-3p mimics, miR-152-3p mimics negative control, miR-152-3p inhibitors and miR-152-3p inhibitors negative control. Bar represents 100 nm.

Relative (c) miR-152-3p and (d) primary miR-152-3p in BMMs cultured with sEVs for 72 hours.


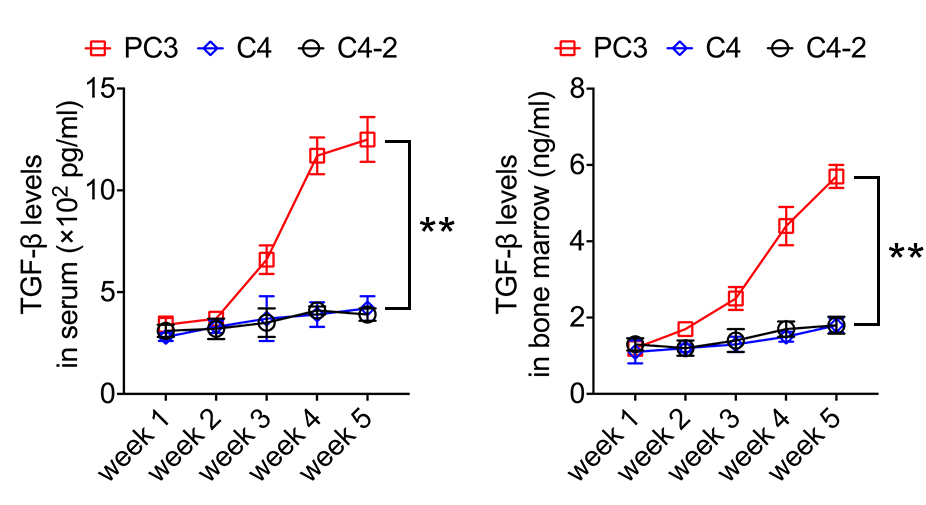


**Supplementary Figure S5. Serum levels of miR-152-3p associate with tumor-induced osteolysis**

ELISA for serum or bone marrow TGF-β concentrations.


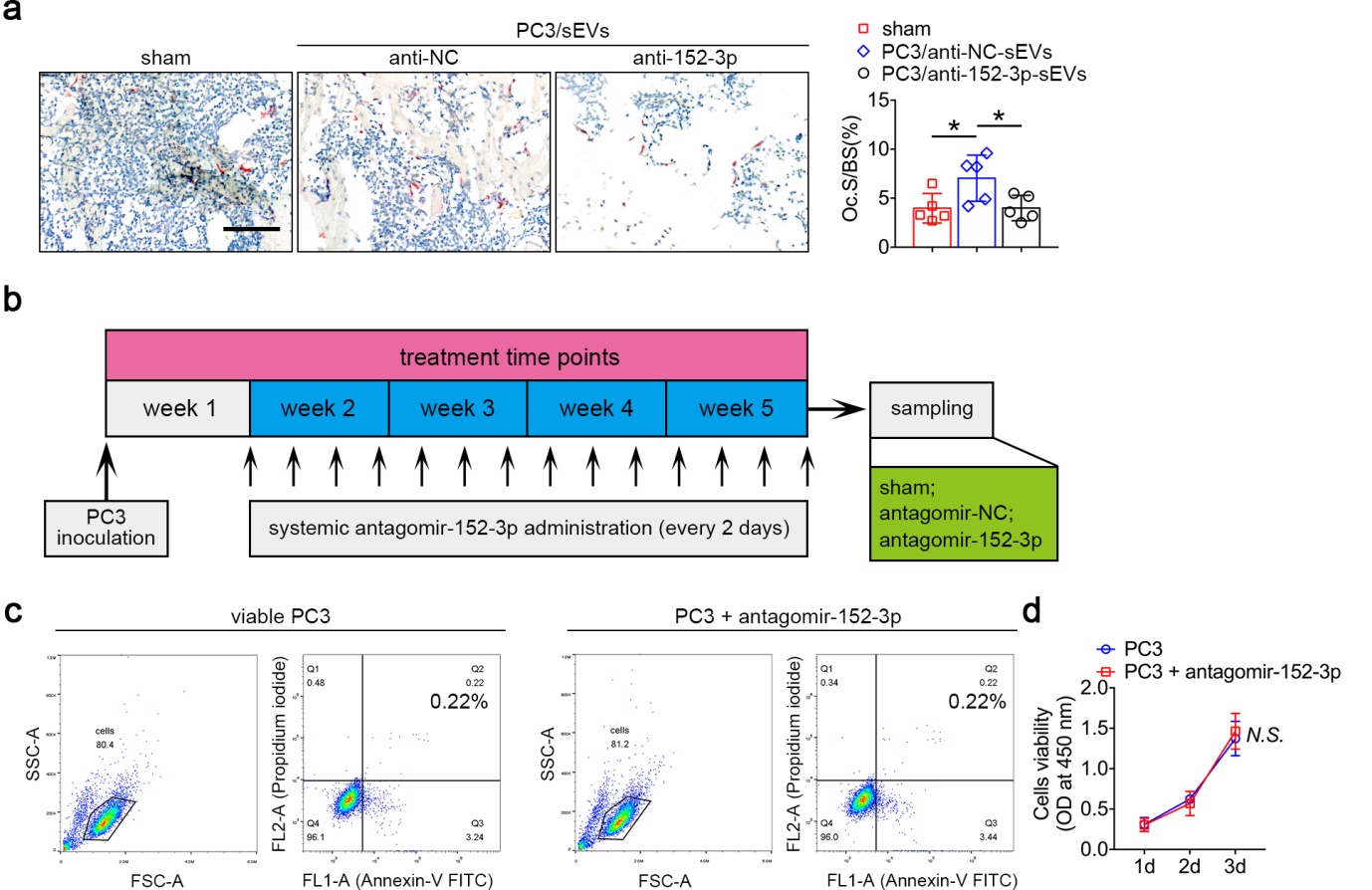


**Supplementary Figure S6. Targeting miR-152-3p to rescue osteolytic progression during tumor growth**

(a) Representative histological TRAP images of tibia sections from mice treated with PBS, PC3/miR-NC-sEVs and PC3/anti-miR-152-3p-sEVs. Bar represents 200 μm. Quantification of osteoclast surface/bone surface in indicated groups, n=5.

(b) Xenograft mice inoculated with osteolytic PC3 were intravenously injected with PBS, antagomir negative control (50 μg/g) and antagomir-152-3p (50 μg/g) every 2 days until the week 5, n=8.

(c) The cytotoxicity of antagomir-152-3p to PC3 was evaluated by flowcytometry.

(d) The viability of PC3 was assessed at 1, 2, and 3 days after treatment with antagomir-152-3p.

Supplementary Table 1. Primer sequences for qPCR

| **Genes** | **Forward** | **Reverse** | **Tm (°C)** |
| --- | --- | --- | --- |
| *Nfatc1* | 5'-GACCCGGAGTTCGACTTCG-3' | 5'-TGACACTAGGGGACACATAACTG-3' | 61 |
| *Ctsk* | 5'-GAAGAAGACTCACCAGAAGCAG-3' | 5'-TCCAGGTTATGGGCAGAGATT-3' | 60 |
| *Acp5* | 5'-GACTTCATCATGTCTCTGGG-3' | 5'-AAAGGTCTCCTGGAACCTC-3' | 58 |
| *Atp6v0d2* | 5'-AGGAAGAAGCTCTGCAGAG-3' | 5'-TAGCTGCATGTCATGTAGGT-3' | 59 |
| *Gapdh* | 5'-AAATGGTGAAGGTCGGTGTG-3' | 5'-TGAAGGGGTCGTTGATGG-3' | 59 |
| *miR-152-3p* | 5'-TCGGCAGGTCAGTGCATGACAGAA-3' | 5'-CTCAACTGGTGTCGTGGA-3' | 60 |
| *RNU-6* | 5'-GAGGGCCTATTTCCCATGATT-3' | 5'-TAATTAGAATTAATTTGACT-3' | 59 |
